# Comparative RNA-seq analysis of *Arabidopsis thaliana* response to *At*Pep1 and flg22, reveals the identification of PP2-B13 and ACLP1 as new members in pattern-triggered immunity

**DOI:** 10.1371/journal.pone.0297124

**Published:** 2024-06-04

**Authors:** Mehdi Safaeizadeh, Thomas Boller, Claude Becker

**Affiliations:** 1 Department of Cellular and Molecular Biology, Faculty of Life Sciences and Biotechnology, Shahid Beheshti University, Tehran, Iran; 2 Zürich-Basel Plant Science Center, Department of Environmental Sciences, University of Basel, Basel, Switzerland; 3 LMU Biocentre, Faculty of Biology, Ludwig-Maximilian-University Munich, Martinsried, Germany; Instituto de Biologia Molecular y Celular de Plantas, SPAIN

## Abstract

In this research, a high-throughput RNA sequencing-based transcriptome analysis technique (RNA-Seq) was used to evaluate differentially expressed genes (DEGs) in the wild type Arabidopsis seedlings in response to *At*Pep1, a well-known peptide representing an endogenous damage-associated molecular pattern (DAMP), and flg22, a well-known microbe-associated molecular pattern (MAMP). We compared and dissected the global transcriptional landscape of *Arabidopsis thaliana* in response to *At*Pep1 and flg22 and could identify shared and unique DEGs in response to these elicitors. We found that while a remarkable number of flg22 up-regulated genes were also induced by *At*Pep1, 256 genes were exclusively up-regulated in response to flg22, and 328 were exclusively up-regulated in response to *At*Pep1. Furthermore, among down-regulated DEGs upon flg22 treatment, 107 genes were exclusively down-regulated by flg22 treatment, while 411 genes were exclusively down-regulated by *At*Pep1. We found a number of hitherto overlooked genes to be induced upon treatment with either flg22 or with *At*Pep1, indicating their possible involvement general pathways in innate immunity. Here, we characterized two of them, namely *PP2-B13* and *ACLP1*. *pp2-b13* and *aclp1* mutants showed increased susceptibility to infection by the virulent pathogen *Pseudomonas syringae* DC3000 and its mutant *Pst* DC3000 *hrcC* (lacking the type III secretion system), as evidenced by increased proliferation of the two pathogens *in planta*. Further, we present evidence that the *aclp1* mutant is deficient in ethylene production upon flg22 treatment, while the *pp2-b13* mutant is deficient in the production of reactive oxygen species (ROS). The results from this research provide new information for a better understanding of the immune system in Arabidopsis.

## Introduction

As sessile organisms, plants are constantly under attack by a broad range of different microbes [1–7]. In a co-evolutionary arms race between plants and pathogens, plants initially sense the presence of microbes by perceiving microbe-associated molecular patterns (MAMPs) via membrane-resident pattern recognition receptors (PRRs) that are located on the cell surface; such MAMP perception generally leads to pattern-triggered immunity (PTI, [1, 4, 8, 10]).

The model plant *Arabidopsis thaliana* can detect a variety of MAMPs, including fungal chitin and bacterial elicitors such as flagellin and elongation factor-Tu (EF-Tu), or their respective peptide surrogates flg22 and elf18 [9–11]. Flagellin and EF-Tu are perceived by FLS2 and EFR receptors, respectively. Besides MAMPs, molecular patterns derived from the plant upon pathogen attack can also trigger an immune response. Examples of such damage-associated molecular patterns (DAMPs) are the AtPeps, a family of endogenous peptide signals released upon cellular damage in *A*. *thaliana*. The different AtPeps (AtPep1-8) originate from the conserved C-terminal portion of their respective precursors AtPROPEP1–8 [12–17]. The plant cell surface PRRs *At*PEPR1 and *At*PEPR2 have been identified as the AtPeps receptors [13, 18, 19].

DAMP/MAMP perception triggers a vast array of defense responses [1, 13, 14]. These include the production of reactive oxygen species (ROS) in an oxidative burst [20, 21], the multi-level specific reprogramming of expression profiles at transcriptional and also post-transcriptional levels [22–25], and downstream defense responses, including callose deposition [26], MAP kinase activation, and synthesis of the defense hormones ethylene and salicylic acid (SA). MAMP treatment prior to the actual pathogen attack results in enhanced resistance to adapted pathogens, and it has been observed that mutants impaired in MAMP recognition display enhanced susceptibility, not only to adapted but also to non-adapted pathogens [11, 21, 25, 27]. This indicates a contribution of pattern-triggered immunity (PTI) to both basal and non-host resistance, highlighting the importance of PTI in plant innate immunity [28–31].

The proteobacterium *Pseudomonas syringae* is a bacterial leaf pathogen that causes destructive chlorosis and necrotic spots in different plant species, including monocots and dicots. *P*. *syringae* pathovars and races differ in host range among crop species and cultivars, respectively [6, 32]. Many strains of *P*. *syringae* are pathogenic in the model plant *A*. *thaliana*, which makes *P*. *syringae* an ideal model to investigate plant-pathogen interactions [32–34].

*P*. *syringae* encodes 57 families of different effectors injected into the plant cell by the T3SS [34]. Effectors inside plant cells are recognized by R proteins, which constitute the second level of defense known as effector-triggered immunity (ETI, [1, 22, 35]). Recently, it was observed that to have an adequate defense response against *P*. *syringae*, the activation of the immune pathways by PTI-activating cell-surface receptors and ETI-activating intracellular receptors is required [36–38]. These studies showed that ETI and PTI potentiate each other and that important components of these two pathways cooperate [36–38]. However, our understanding of the interconnection between these two pathways remains incomplete.

PTI response is controlled by a complex, interconnected signaling network, including many transcription factors (TFs); interference with this network can paralyze the adequate response upon pathogen infection [39–42]. Launching effective and robust PTI requires the activation of specific TFs as a consequence of defense signal perception [41–44]. The major specific defense TFs are regulated by MAPK cascade factors such as WRKY22, WRKY29, and WRKY33 [40–42]. Recent studies showed that WRKYs often form positive feedback regulatory loops in defense signal perception [42, 43]. Furthermore, there are other TFs that are regulated by Ca^2+^ signaling [41, 44]. Calmodulin binding protein 60g (CBP60g) is the well-identified TFs that are regulated by Ca^2+^ signaling [43, 44]. Recent studies showed that CBP60g directly binds to the promoter regions of key genes which have a role in innate immunity [41, 43, 44]. In addition to specific reprogramming of transcription, post-transcriptional regulation also plays a role in the plant immune response [45]. The activation of TFs is not limited to the microbial infection; using cap analysis of gene expression, it was recently observed that as a consequence of flg22 perception, a cluster of genes rapidly and transiently induced by MAMPs is enriched in TFs [41, 46]. The advent of advanced sequencing and proteomics technologies has led to the identification of many novel players in defense signaling pathways and their characterization as important components of innate immunity in *Arabidopsis*. However, for a fundamental understanding of the plant’s defense system and its response to pathogens, it is necessary to fill the remaining gaps by further identifying genes and proteins involved in plant immunity [1].

Studies showed that both the flg22 (a well-known MAMP) and AtPep1 (the best-studied member of the DAMP family of AtPeps) trigger immunity in *A*. *thaliana* [8, 12]. The highly conserved 22-amino-acid fragment (flg22) of bacterial flagellin that is recognized by the FLS2 PRR can activate an array of immune responses in Arabidopsis [1–4]. Similarly, AtPep1 perception by AtPEPR1/2 can activate the immune system in Arabidopsis. In addition, resistance to *Pst* DC3000 is induced by pre-treatment with flg22 [1–4, 10, 25]. Considering flg22 as the exogenous defense signal and AtPep1 as the endogenous defense signal that activate the immune system in Arabidopsis, we set out to analyze and compare their respective effects side by side in one coherently designed experiment, hoping that this would allow to detect shared features and specific responses of the respective immune response pathways. Furthermore, previous studies investigating flg22-induced transcriptional changes showed that among highly induced genes, there were several ones with functions in innate immunity pathways in Arabidopsis [8, 25, 46–48]. We speculated that a whole-genome transcriptome profiling analysis of elicitor-treated Arabidopsis plants would unveil additional new players in the immune signaling system.

Here, we performed whole-genome transcriptome profiling by RNA sequencing (RNA-seq) [49–52] of Arabidopsis seedlings treated with either flg22 or AtPep1 treatments. Filtering for genes induced in both treatments and those missing in previously published assays, we selected 85 candidate genes to be investigated for their role in plant immune response and systematically tested T-DNA insertion mutants of these genes for susceptibility towards *Pst*. For two loci, *PHLOEM PROTEIN 2-B13* (*PP2-B13*) and *ACTIN CROSS-LINKING PROTEIN 1* (*ACLP1*), we identified mutant lines with altered pathogen response phenotypes and characterized these genes as novel players in early PTI responses.

## Materials and methods

### Plant material and growth conditions

All Arabidopsis genotypes were derived from the wild-type accession Columbia-0 (Col-0). The plants were grown as one plant per pot at 10 h photoperiod light at 21°C and 14 h dark at 18°C, with 60% humidity for 4 to 5 weeks, or were grown on plates containing Murashige and Skoog (MS) salts medium (Sigma, Aldrich), 1% sucrose, and 1% agar with a 16 h photoperiod. Seeds of the *sid2* mutant line were kindly provided by Jean-Pierre Métraux (University of Fribourg). The *fls2* mutant line was previously published [25]. *pp2-b13 (*AT1G56240; SALK_144757.54.50), and *aclp1* (AT1G69900; SALK_68692.47.55) were obtained from the Nottingham Arabidopsis Stock Centre (NASC). Experimental research on *A*. *thaliana* seeds and plants that were investigated in this study were handled according to methods recommended by Arabidopsis Biological Resource Center (ABRC; https://abrc.osu.edu/) and NASC under the guidelines considering all relevant rules and legislations to study the Arabidopsis mutant lines. Furthermore, we had verbal consent of the Institute to study Arabidopsis mutants.

### Peptide treatments

The peptides used as elicitors were flg22 (QRLSTGSRINSAKDDAAGLQIA), and *At*Pep1 (ATKVKAKQRGKEKVSSGRPGQHN). The peptides were ordered from EZBiolabs (EZBiolab Inc., IN, USA), dissolved in a BSA solution (containing 1 mg/mL bovine serum albumin and 0.1 M NaCl), and kept at -20°C. In order to prepare sterile seedlings, Arabidopsis seeds were washed with 99% ethanol supplemented with 0.5% Triton for 1 min, washed with 50% ethanol supplemented with 0.5% Triton for 1 min, then washed with 100% ethanol for 2 min. Seeds were sown on MS salt medium supplemented with 1% sucrose and 0.8% Phytagel (Sigma-Aldrich) at pH 5.7. Subsequently, the plates were stratified for 2 d at 4°C and germinated at 21°C at 10 h photoperiod light and 14 h dark (MLR-350; Sanyo chamber). One day before treatment, the seedlings were moved from plates to ddH_2_O. One-week-old Arabidopsis seedlings were treated with *At*Pep1 and flg22 (1 μM) for 30 min. BSA solution was used for the mock-treated control.

### RNA isolation, Illumina sequencing, and quality control

Total RNA was isolated from one-week-old Arabidopsis seedlings using the RNeasy Plant Mini Kit (Qiagen), according to the manufacturer’s protocol. Three individual biological replicates were used per condition. RNA purity, concentration, and integrity were first determined via spectrophotometric measurement on a NanoDrop 2000 (Thermo-Scientific) and subsequently was measured using Bioanalyzer (Thermo-Scientific) to determine RIN score. Libraries were prepared using the RNA sample preparation kit (Illumina) according to the manufacturer’s instructions (Illumina). Libraries were sequenced on a HiSeq2000 instrument (Illumina) as 100 bp single-end reads. The sequencing quality of the fastq files from the RNA-Seq data was examined by FastQC software (version V0.10.1; http://www.bioinformatics.babraham.ac.uk/projects/fastqc/). Adapter sequences were clipped and low-quality reads were either trimmed or removed.

### Mapping reads to the reference genome and analysis of differentially expressed genes (DEGs)

RNA-seq reads were aligned against the *A*. *thaliana* cDNA reference genome (TAIR10; (https://www.arabidopsis.org/). The reference genome index was constructed with Bowtie v2.2.3 and reads were aligned to the Arabidopsis reference genome using TopHat v2.0.12 with default parameters [53]. The detailed information of Illumina sequencing data and mapped read is presented in the S1 Table. The resulting alignments were visualized using Integrative Genomics Viewer (IGV, [54]). To evaluate differentially expressed genes between elicitor-treated and control samples, we used the DESeq2 R package [55, 56]. Genes with an adjusted *p*-value < 0.05 and a minimum two-fold change in expression were considered as differentially expressed.

### Selection of candidate genes

Because we were interested in genes not yet classified as related to immune response, we applied several filters: from the genes significantly up-regulated after 30 minutes of flg22 or AtPep1 treatment, we discarded those which had previously been reported as differentially regulated and implicated in biotic and abiotic stress response [25, 46–48]. We selected a subset of 85 genes (S2 Table) based on the following criteria: 1) high induction of transcription in response to both flg22 and AtPep1 treatments, 2) not present on Affymetrix ATH-22k microarray chips, 3) no published function or at least not connected to defense, and 4) not a member of a large gene family (in order to avoid potential functional redundancy). From this list, we eventually selected 20 genes as candidate genes for further analyses (Table 1 and S1 Fig) and ordered corresponding T-DNA insertion lines (http://signal.salk.edu/cgi-bin/tdnaexpress) from NASC (www.arabidopsis.info).

**Table 1:** Detailed information on the top up-regulated genes based on RNA-seq analysis (fold change 30 minutes after flg22 and AtPep1 treatments compared to the control). Genes of interest are highlighted in bold.

Accession Number	flg22 treatment		AtPep1 treatment		Putative function of the gene	Gene location	T-DNA insertion mutant/NASC Code	Final Genotyping results confirmed by PCR and consideration for subsequent study
	Fold change	log_2_	Fold change	log_2_				
AT5G11140	503	8.97518	141	7.14031	Encodes an Arabidopsis phospholipase-like protein (PEARLI 4) family	Chr5:3545211–3546169	Not available	Not considered
**AT1G56240**	**126**	**6.97733**	**120**	**6.91095**	**Encodes a phloem protein 2-B13 ("PP2-B13"); function in: carbohydrate binding; F-box domain, cyclin-like, F-box domain, Skp2-like**	Chr1:21056099–21057577	**detected/SALK_144757.54.50**	**Homozygous line; “Considered”**
AT2G32200	95	6.57146	36	5.16218	Encodes an unknown protein	Chr2:13676389–13677306	—	Not considered
AT1G05675	72	6.17964	63	5.97188	Encodes an UDP-Glycosyltransferase superfamily protein	Chr1:1701116–1702749	—	Not considered
**AT1G65385**	**65**	**6.01185**	**39**	**5.2804**	**Encodes an pseudogene, putative serpin**	Chr1:24289566–24291055	**detected/N570388, SALK_070388**	**Homozygous line; “Considered”**
AT4G18195	60	5.9082	46	5.50896	Encodes the protein which is the member of a family of proteins related to PUP1, a purine transporter	Chr4:10069458–10071115	—	Not considered
AT5G36925	53	**5.72585**	55	5.78087	Encodes a protein with unknown protein	Chr5:14565476–14566439	—	Not considered
AT1G61470	33	5.03187	21	4.38478	Encodes a polynucleotidyl transferase protein which is, ribonuclease H-like superfamily protein	Chr1:22678092–22679302	—	Not considered
**AT4G23215**	**30**	4.9223	**28**	**4.83246**	**Encodes a pseudogene of cysteine-rich receptor-like protein kinase family protein pseudogene**	Chr4:12152900–12153459	**detected/N605169, SALK_105169**	**Homozygous line; “Considered”**
AT5G09876	29	4.83977	11	3.4823	Encodes an unknown protein	Chr5:3079887–3080435 Chr1:22032313–22033297	—	Not considered
AT1G59865	28	4.79972	39	5.27729	Encodes an unknown protein	Chr1:22032313–22033297	detected/N584779, SALK _084779	Not detected; Not considered
AT2G35658	28	4.79818	22	4.46888	Encodes an unknown protein	Chr2:14990325–14990935	detected/N825604, SAIL_600_D01	Not detected; Not considered
**AT1G24145**	**26**	4.71633	**11**	**3.52029**	**Encodes an unknown protein, located in: endomembrane system**	Chr1:8540838–8542053	**detected/N835081, SAIL_784_C07**	**Homozygous line**; Not considered
AT3G07195	24	4.58759	19	4.28252	Encodes a RPM1-interacting protein 4 (RIN4) family protein	Chr3:2288732–2290515	—	Not considered
AT1G18300	22	4.48140	9	3.15319	Encodes a nudix hydrolase homolog 4 (NUDT4) protein	Chr1:6299669–6301139	—	Not considered
AT2G24165	22	4.47869	16	4.03998	Encodes a pseudogene, similar to HcrVf3 protein	Chr2:10272672–10273595	—	Not considered
**AT1G69900**	**20**	**4.34832**	**10**	**3.3191**	**Encodes an actin cross-linking protein; CONTAINS InterPro DOMAIN/s ("ACLP1")**	Chr1:26326216–26327965	**detected/N568692, SALK_068692 (AR)**	**Homozygous line**; Not considered
**AT2G27389**	**20**	**4.288968**	**13**	**3.715253**	**Encodes an unknown protein**	**Chr2:11720294–11721081**	**detected/SALK_142825.23.95**	**Homozygous line**; Not considered
AT4G39580	18	4.16251	21	4.40726	Encodes a Galactose oxidase/kelch repeat superfamily protein	Chr4:18385684–18386811	—	Not considered
**AT1G30755**	**14**	**3.83941**	**13**	**3.74068**	**Encodes an unknown protein**	**Chr1:10905731–10909760**	**Not detected/N666232, SALK_063010C**	**Not detected**; Not considered

### Determination of gene expression by quantitative real-time reverse transcription PCR analysis

To confirm the reliability of the RNA sequencing results, the expression of the 20 selected up-regulated genes in One-week-old Arabidopsis seedling in response to flg22 and AtPep1 treatments, was measured and validated by q-reverse transcription PCR. For treatment, 1 μM flg22 and 1uM AtPep1 dissolved in BSA solution (1 mg/mL bovine serum albumin and 0.1 M NaCl). BSA solution without any elicitor was used for the mock-treated control. 30 minutes after treatment, seedlings were removed, immediately were put in liquid nitrogen and total RNA were extracted using BioFact^™^ (South Korea) RNA extraction kit. The specific primers that were used in these experiments are listed in the S3 Table. *Actin1* (*At2g37620*), *Actin2* (*At3G18780*) or *Ubiquitin* (*AT4G05320*) transcript levels were used as internal reference [57, 58]. Furthermore, to determine the gene expression of *At1G56240* and *At1G69900* in the mature plants, discs of leaves of four-week-old Arabidopsis plants were cut out using a sterile cork borer (d = 7mm) and placed overnight in ddH_2_O in a 5 cm Petri dish. Thereafter, the experiment started (time zero) with the addition of 1 μM flg22 and 1uM AtPep1 dissolved in BSA solution (1 mg/mL bovine serum albumin and 0.1 M NaCl). BSA solution without any elicitor was used for the mock-treated control. In order to produce a time-course in response to flg22 treatment and AtPep1 treatment, the experiment was stopped after 30 min, 2 h, and 6 h. Total RNA from leaf discs of four-week-old Arabidopsis plants was extracted using the NucleoSpin RNA plant extraction kit (Macherey-Nagel) and treated with DNase according to the manufacturer’s extraction protocol. RNA quality of all samples was assessed using NanoDrop 2000 (Thermo-Scientific). To synthesize the cDNA, 10 ng of RNA was used with oligo (dT) primers, and AMV reverse transcriptase, and reverse transcription was performed according to the manufacturer’s instructions (Promega). Using a GeneAmp 7500 Sequence Detection System (Applied Biosystems), quantitative RT-PCR was performed in a 96-well format. The gene-specific primers used in this study are listed in the S3 Table. The expression of *UBQ10* (*AT4G05320*), which has been validated for gene expression profiling upon flg22 treatment [59, 60], was used as the reference gene. Based on C_T_ values and normalization to *UBQ10* (*AT4G05320*) expression, the expression profile for each candidate gene was calculated using the qGene protocol [59–61].

### Analysis of T-DNA insertion mutants

After grinding leaf material in liquid nitrogen, total DNA was extracted using EDM-Buffer (200 mM Tris pH7.5; 250 mM NaCl, 25 mM EDTA; 0.5% SDS). Putative T-DNA insertion mutants were genotyped by PCR. We designed gene-specific primer pairs LP and RP based on the predicted genomic sequence surrounding the T-DNA insertion (S3 Table). The plants were considered homozygous mutants if there was a PCR product with T-DNA-specific border primers LP/ LBa1 but not with the LP/RP primers (S3 Table). The PCR products were visualized and photographed with UV-illuminator Bio-Rad gel doc using Quantity One imaging software (Bio-Rad, USA). We obtained T-DNA insertion mutants of six single homozygous lines bearing a disruption in the gene, including *AT1G56240* (*PP2-B13*) and *AT1G69900* (*ACLP1*; Table 1).

### RT-PCR experiment

For total RNA extraction, samples of leaf tissue from 4-week-old Arabidopsis including wild type plants (Col-0), *pp2-b13*, and *aclp1*were harvested into liquid nitrogen and were ground with a sterile mortar and pestle. The NucleoSpin RNA Prep Kit (BioFACT^™^, South Korea) was used for RNA extraction according to the manufacturer’s instructions and DNase-treated. Reverse transcription was performed at 50°C for 45 minutes using total RNA, a reverse transcriptase (BioFACT^™^, South Korea) and an oligo (dT)20 primer (BioFact, South Korea) supplemented with 0.5 μl RNase inhibitor (BioFACT^™^, South Korea) and according to the manufacturer’s instructions. To ensure the specificity and accuracy of each primer and to design the highly specific primers for *PP2-B13* and *ACLP1* transcripts, the oligonucleotide primers were designed by the AtRTPrimer program [62] which exclusively determines specific primers for each individual transcript in Arabidopsis. The housekeeping gene *ACTIN2* was used as a positive control for each PCR. The primers for *ACTIN 2* transcript were used as described previously [63]. Primers that were used in these experiments are listed in the S3 Table. The RT-PCR products were visualized and photographed with UV-illuminator Proxima 10phi gel doc using Proxima AQ-4 imaging software (ISOGEN, Netherland).

### Bacterial growth assay

*Pseudomonas syringae* DC3000 *hrcC-* mutant (deficient in type three effector secretion system, [64, 65]); and *Pst* DC3000 were grown in 20 ml liquid YEB medium supplemented with 50 μg/ml Rifampicin on a shaker at 28°C overnight. Infection assay and counting of the bacterial titer were done as described previously [66] with a bacterial suspension at OD_600_ = 0.0002. Leaves of 4-5-week-old Arabidopsis plants were infiltrated using a syringe. The *sid2-2* mutant plants, which are incapable of accumulating salicylic acid [67], were used as a positive control. Mock-infected plants were similarly treated with infiltration buffer.

### Measurement of ethylene production

Ethylene production was measured as described previously [68] with the exception that six-leaf strips were placed together in a 6 ml glass vial containing 0.5 ml of ddH_2_O.

### ROS measurement

Reactive oxygen species generation was measured as described previously [59]. Using a plate reader (MicroLumat LB96P, Berthold Technologies) light emission was determined over 30 min, starting from the addition of the elicitor.

### Immunoblot analysis

A sample of leaf material (150 mg) from 4-5-week-old Arabidopsis plants was shock-frozen and ground in liquid nitrogen. 200 μl Läemmli buffer containing 50 mM β-mercaptoethanol was added and the ground homogenate was further mixed by vortexing. Proteins were denatured by boiling for 10 min at 95°C. Debris was pelleted by centrifugation for 5 min at 13,000 rpm. Total proteins were separated by electrophoresis in 7% SDS-polyacrylamide gels and electrophoretically transferred to a polyvinylidene fluoride membrane according to the manufacturer’s protocol (Bio-Rad, U.S.A). Transferred proteins were detected with Ponceau-S. The abundance of FLS2 receptor was analyzed by immunoblot and immunodetection with anti-FLS2-specific antibodies as previously described [68] and the western signals were visualized on an Azure c600 Imager (Azurebiosystems).

### Determination of phytohormone salicylic acid levels

*Pseudomonas syringae* DC3000 *hrcC-* was cultured in 20 ml liquid YEB medium supplemented with 50 μg/ml Rifampicin on a shaker at 28°C overnight. The overnight liquid cultures were diluted to a concentration to an optical density at 600 nm (OD600) = 0.0002. Using a needleless syringe, the diluted bacteria were injected into leaves of four-five weeks old *pp2-b13*, and *aclp1* and wild-type plants. Fours leaves for each plants were infiltrated. Treated plants with infiltration buffer regarded as Mock-Control. Six plants were used for each replicates and three replicates were taken for each experiments. 48-hour post infiltration the leaves were collected and the free SA levels was measured as described previously [69]. The experiment was repeated for two times. Statistical analyses were performed using the Students *t*-test.

### Phylogenetic analysis and comparison consensus of the amino acid sequences

Protein sequences BB2-B13 and ACLP1 were used as queries using BLASTP (Basic Local Alignment Search Tool, [70]) search to identify the most similar proteins in *A*. *thaliana* and diverse land plants. We applied a cutoff of 70%≤ sequence identity on the top hit of the BLASTP search for BB2-B13 and ACLP1 and their orthologous and prologues were identified. Protein sequences with more than 70% sequence identity were downloaded from the NCBI database and multiple sequence alignment were performed based on the ClustalW software [71]. Phylogenetic analyses and graphical representation were carried out using MEGA software (Molecular Evolutionary Genetics Analysis) version 6.0 [72]. A neighbor-joining phylogenetic tree was constructed after 1,000 iterations of bootstrapping of the aligned sequences. All branches with bootstrap values <60% were collapsed. To compare the consensus of the amino acid sequences, sequence logos were generated using WebLogo (http://www.weblogo.berkeleky.edu/), using the ClustalW alignment as input.

## Results

### Whole-genome transcriptional profiling identifies two novel factors of PTI

**Fig 1:**
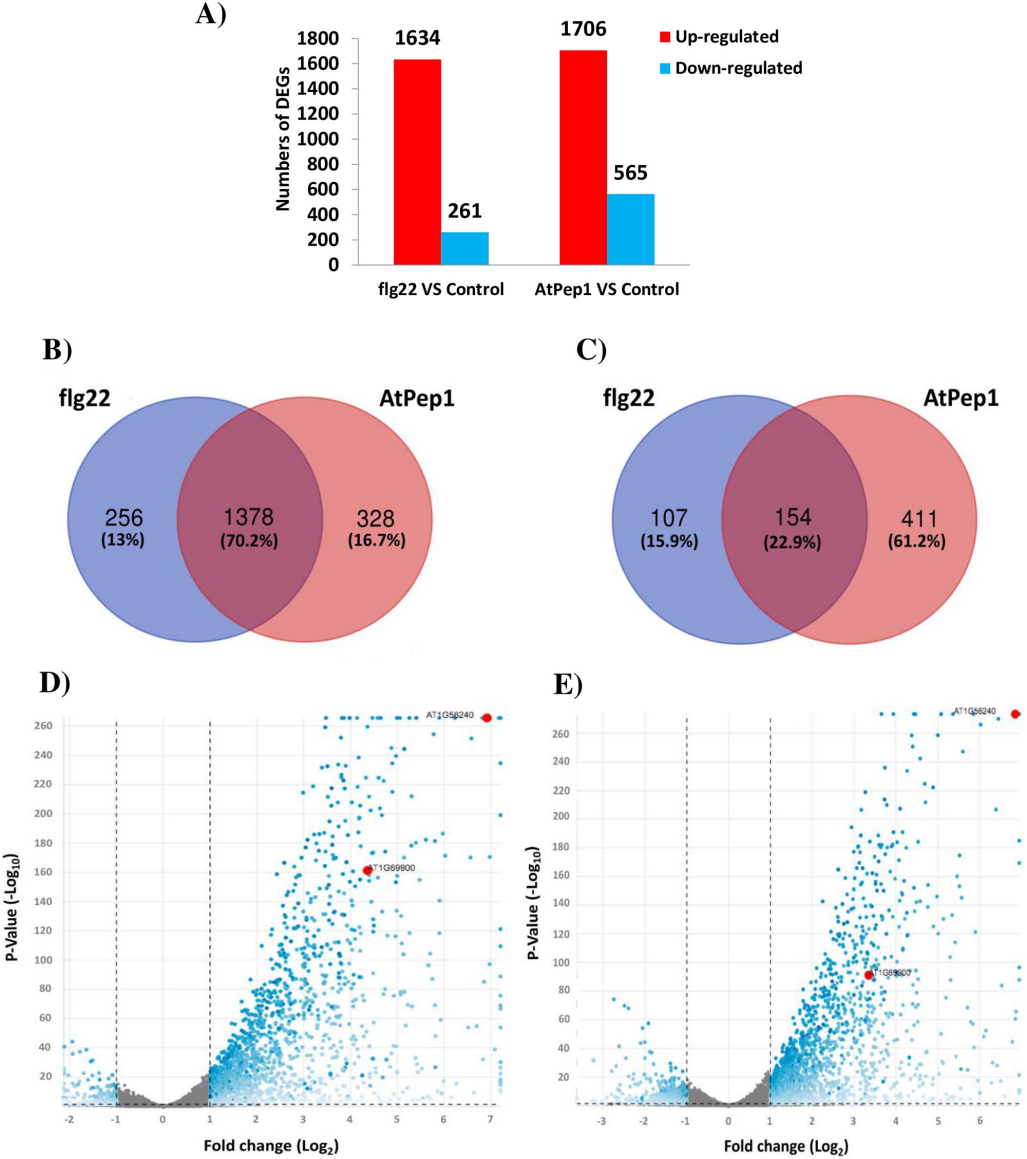
Distribution of differentially expressed genes (DEGs). (A) DEGs in *Arabidopsis thaliana* in response to flg22 and AtPep1 treatments compared to the control investigated in this study. Red bars correspond to the up-regulated genes; blue bars correspond to the down-regulated genes. (B) Venn diagram of up-regulated DEGs between flg22 treatment and AtPep1 treatments. (C) Venn diagram of down-regulated DEGs between flg22 treatment and AtPep1 treatments. In B and C, the overlapping regions display the common transcripts. (D) Volcano plot of DEGs in response to flg22 treatment; (E) Volcano plot of DEGs in response to AtPep1 treatment. In (D-E), blue dots correspond to significantly up- and down-regulated DEGs, grey dots represent non-DEGs. *At1G56240* (*PP2-B13*) and *At1G69900* (*ACLP1*) are highlighted in red.

To dissect transcriptional responses in responses in response to flg22 and AtPep1, we extracted total RNA from mock- and elicitor-treated one-week-old Arabidopsis plants and performed RNA-seq transcriptome analysis on three biological replicates per treatment. Samples were collected 30 min after elicitor treatment. We used the R package DESeq2 [55] for differential gene expression analysis (a complete list of all genes in response to flg22 treatment and AtPep1 treatments are in S4 and S5 Tables, respectively). In response to flg22, we detected a total of 1,895 DEGs compared to the control treatment (Fig 1A), of which 1,634 genes were up and 261 were down-regulated in the flg22-treated seedlings (a complete list of all DEGs up-regulated by flg22 is found in S6 Table, a list of all DEGs down-regulated by flg22 is found in S7 Table). Treatment with AtPep1 resulted in 2,271 DEGs, with 1,706 up-regulated and 565 down-regulated (Fig 1A; a complete list of all DEGs up-regulated by AtPep1 is found in S8 Table, a list of all DEGs down-regulated by AtPep1 is found in S9 Table). When comparing the two treatments with each other, we detected only 511 DEGs, with similar fractions of up and down-regulated genes (265 and 246, respectively, in flg22 vs. AtPep1; Fig 1A); a complete list of all up-regulated DEGs in response to flg22 treatment compared to the AtPep1 treatment is found in S10 Table; a list of all DEGs down-regulated in response to flg22 treatment compared to the AtPep1 treatment is found in S11 Table). Taken together, these results indicated that AtPep1 treatment causes slightly more genes to be differentially regulated than flg22, and that the transcriptional profiles are more similar between flg22 and AtPep1-treated samples than between either of the treatments and the control. While a remarkable 70% of flg22 up-regulated genes were also induced by AtPep1, 256 genes were exclusively up-regulated in response to flg22, while 328 were exclusively up-regulated in response to AtPep1 (Fig 1B; a complete list of all DEGs exclusively up-regulated in response to flg22 treatment is found in S12 Table, a complete list of all DEGs exclusively up-regulated in response to AtPep1 treatment is found in S13 Table). Of genes down-regulated upon flg22 treatment, only 23% were also down-regulated in response to AtPep1; 107 genes were exclusively down-regulated by flg22 treatment, vs. 411 genes by AtPep1 (Fig 1C; a complete list of all DEGs exclusively down-regulated in response to flg22 treatment is found in S14 Table, a complete list of all DEGs exclusively down-regulated in response to AtPep1 treatment is found in S15 Table).

Former studies showed that treatment of Arabidopsis seedlings with flg22 triggers robust PTI-like responses at the transcriptional level, activating ca. 1,000 genes that may have functions in PTI responses [25, 46–48]. However, because these experiments were done using the ATH1 microarray, which does not cover all Arabidopsis protein-coding genes, we speculated that there might be additional, so far unknown PTI-related genes affected by flg22 and other elicitors. Denoux et al. [48] performed a comprehensive microarray (Affymetrix ATH1) transcript analysis in response to flg22 treatment. A comparison of the up-regulated DEGs results in RNA-seq experiment analysis with fold change cutoff (adjusted *p*-value < 0.05 and a minimum two-fold change) among the genes which are also present in ATH1 Affymetrix GeneChip showed that 1,366 up-regulated DEGs are present in both RNA-seq experiment and ATH1 Affymetrix GeneChip (S16 Table). Our analysis showed that 268 genes with fold change cutoff (adjusted *p*-value < 0.05 and a minimum two-fold change) are exclusively up-regulated in RNA-seq analysis which were not present in ATH1 Affymetrix GeneChip and their expression was only investigated in RNA-seq analysis (S17 Table). To identify yet unknown PTI factors, we first discarded all genes from our list of DEGs that had been present on the ATH1 microarray chip and hence would have been detected in the above-mentioned studies.

**Fig 2:**
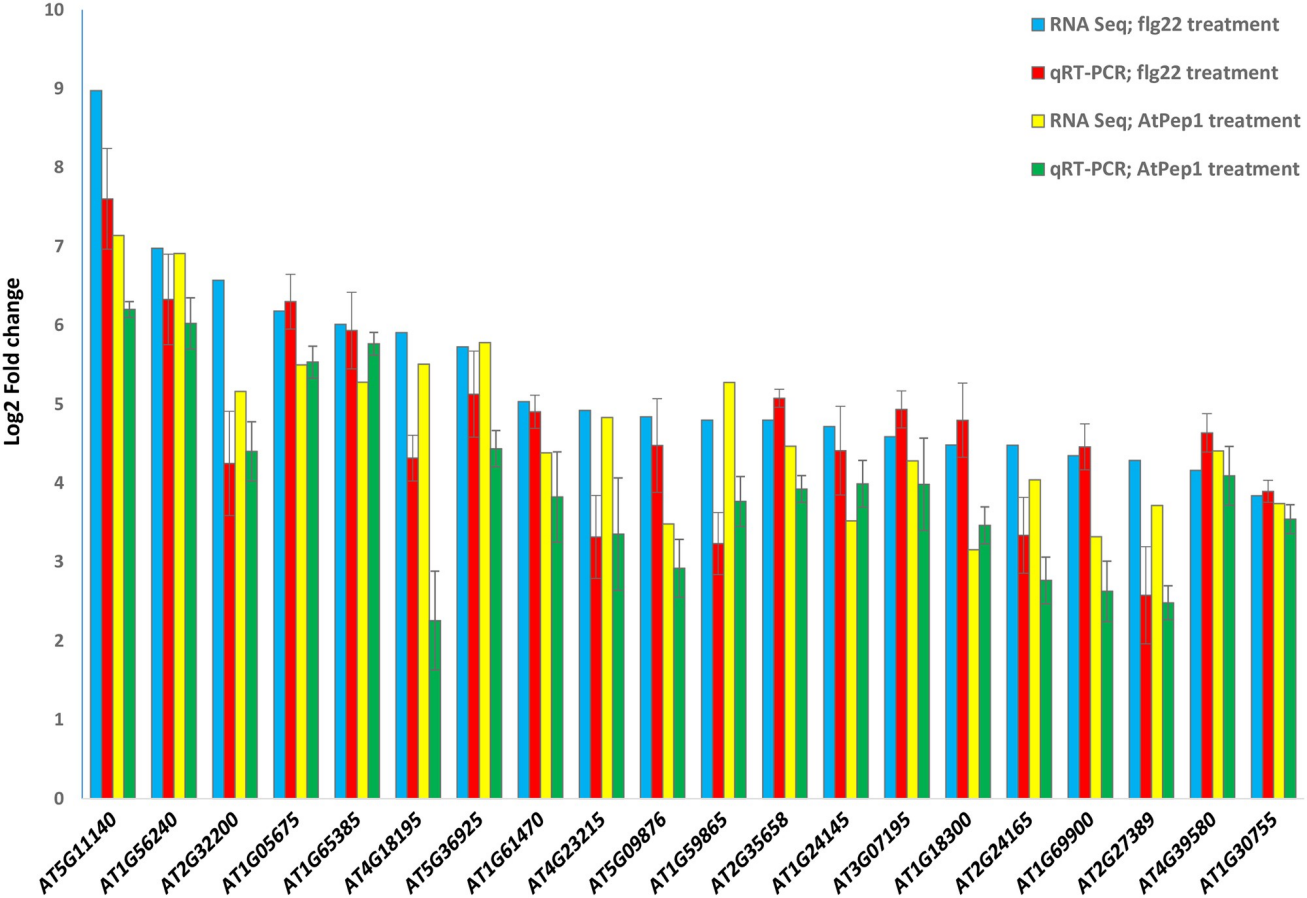
Confirmation of RNA-seq results by quantitative real-time reverse transcription PCR analysis. The expression levels of 20 candidate genes in the RNA-Seq transcriptional profile were validated by qRT-PCR using gene-specific primer sets (S3 Table). One-week-old seedlings of Arabidopsis Col-0 plants were treated with 1 μM flg22 and AtPep1 and the expression patterns of the 20 selected candidates were measured 30 minutes after elicitor treatment. The X-axis indicates the genes name; the Y-axis indicates the log_2_ scale of the gene expression levels. Each bar represents the fold changes relative to mock samples. The relative expression of each gene was normalized to that of *ACTIN2* expression. Values were obtained from the means ± SD of three technical replicates. Two independent experiments were performed with the similar results.

We then ranked the remaining DEGs by fold change induction (high induction of transcription in response to both flg22 and AtPep1 treatments) and selected the 85 most strongly up-regulated genes as candidates (S2 Table). Finally, we decided to focus on a small set of genes that showed the highest induction after flg22 treatment (Table 1). The expression of these 20 candidates in response to flg22 and AtPep1 treatments were checked and RNA-Seq results were validated by quantitative RT-PCR (Fig 2). As can be seen in the Fig 2, in response to flg22 and AtPep1 treatments, the expression of these 20 candidate genes is up-regulated in both RNA-seq analysis and quantitative RT-PCR. In the next step, we checked for the availability of T-DNA insertion mutants for these genes and retrieved mutant lines for AT1G56240, AT1G65385, AT4G23215, AT1G59865, AT1G24145, AT2G35658, AT1G69900, AT2G27389, and AT1G30755. We confirmed homozygous T-DNA insertions via PCR.

### Increased susceptibility to *Pseudomonas syringae* DC3000 *hrc*C- and *Pst* DC3000 in *pp2-b13* and *aclp1* mutant lines

To test whether any of the candidate genes might play a role in immunity and to evaluate their response to bacterial infection compared to the wild-type plants, we tested all homozygous T-DNA mutant lines for bacterial growth of the mutant pathogenic strain *P*. *syringae* DC3000 *hrcC-* mutant (*Pst* DC3000 *hrcC-*), which is defective in T3SS and *P*. *syringae* DC3000.

**Fig 3:**
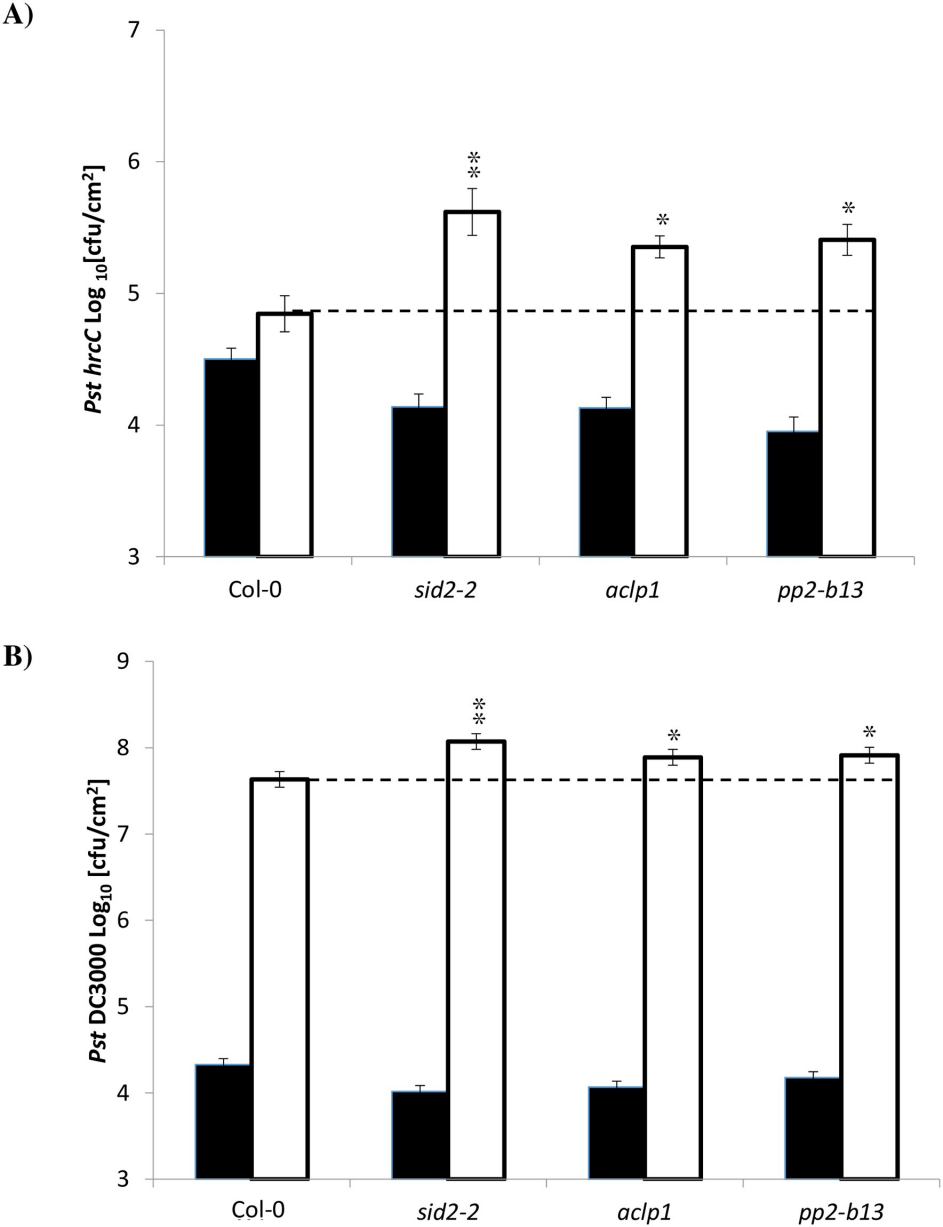
Bacterial susceptibility assay. (A) Leaves of four- to six-week-old Arabidopsis plants (Col-0, *sid2-2*, *pp2-b13*, and *aclp1*) were pressure infiltrated with *Pseudomonas syringae* DC3000 *hrc*C- mutant (OD_600_ = 0.0002, in infiltration buffer). (B) Leaves of four- to six-week-old Arabidopsis plants (Col-0, *sid2-2*, *pp2-b13*, and *aclp1*) were pressure infiltrated with *Pseudomonas syringae* pv. *tomato* DC3000 (OD_600_ = 0.0002, in infiltration buffer). *sid2-2* mutant plants, which are deficient in salicylic acid production, were used as a positive control. Black bars indicate the number of bacterial colony from leaf discs of infected leaves just after infiltration (0 day); white bars represent colony-forming units (cfu/cm^2^) 48 h post-inoculation. Bars show the mean ± s.e. of six technical replicates. Six plants were used for each line. Similar results were observed in four independent experiments. Asterisks indicate a significant difference (*p-value ≤0.05, **p-value ≤0.01) from the wild-type plants as determined by Student’s *t-test*.

Two days post-inoculation of leaves with *Pst* DC3000 *hrcC-*, the bacterial titer for wild type Arabidopsis reached 109,000 cfu/cm^2^, while for *pp2-b13* mutant lines it increased significantly to 325,000 cfu/cm^2^ (*p*-value = *0*.0261), albeit not as drastically as that of *sid2-2* mutants (Fig 3A). As can be seen in Fig 3A, 48 h post-inoculation of leaves with *Pst* DC3000 *hrcC-*, the bacterial titer for wild type Arabidopsis reached 109,000 cfu/cm^2^ while for the *aclp1* mutant line, it was increased significantly at 257,000 cfu/cm^2^ (*p*-value = 0.0089).

As can be seen in Fig 3B, two days post inoculation, the titer for *pp2-b13* mutant lines was 104,000,000 cfu/cm^2^, which was statistically significantly different (*p*-value = 0.0205) compared to wild-type plants. As shown in Fig 3B, 48 h post-inoculation of leaves with *P*. *syringae* DC3000, the titers of *Pst* DC3000 in wild type Arabidopsis leaves reached 52,100,000 cfu/cm^2^, while in the studied mutant lines, the bacterial counts for *aclp1* mutant plants was 104,000,000 cfu/cm^2^, which is roughly double the number of bacteria counted in Arabidopsis wild type plants and, determined using a Student’s t-test, was statistically significantly different (*p*-values 0.0336) compared to the wild type plants.

The protein encoded by *PP2-B13* is a phloem protein containing the F-box domain Skp2. It also has a described function in carbohydrate-binding [73]. The protein encoded by *ACLP1* is of unknown function, with the highest similarity to actin cross-linking proteins, and includes a fascin domain. In conclusion, comparing the bacterial growth titer in the mutant plants to that of wild-type Col-0 revealed that two of the lines, namely SALK_144757.54.50 and SALK_68692.47.55, showed significantly better bacterial growth (*p*-value = 0.0261 and 0.0089, respectively; Student’s *T*-test, Fig 3A) and their underlying loci might play a role in defense signaling. These results suggest that PP2-B13, and ACLP1 are required for wild-type levels of resistance against *P*. *syringae* DC3000 and *Pst* DC3000 *hrcC-*. The symptom of infected plants in *pp2-b13* and *aclp1* mutants compared to wild-type Arabidopsis, two days post-infection with *Pst* DC3000 *hrcC*, is illustrated in S5 Fig. The results of infection assay with *Pst* DC3000 *hrcC* on other mutant lines which were investigated in this study are presented in S6 Fig.

### Expression of the *PP2-B13* and *ACLP1* genes is induced following flg22 and AtPep1 treatment

**Fig 4:**
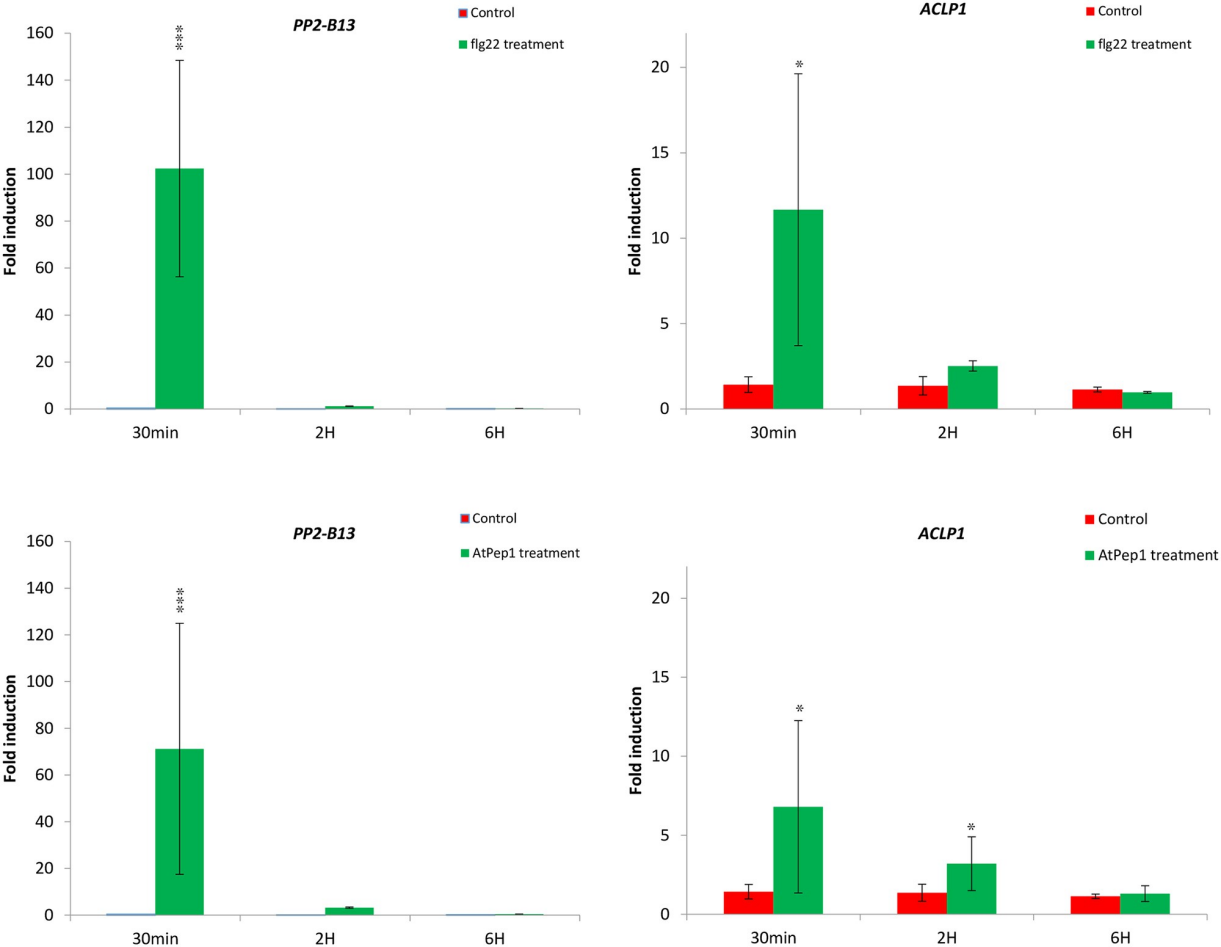
Changes in expression levels of the *PP2-B13* and *ACLP1* genes after elicitor treatment. Leaf discs of five weeks old Arabidopsis Col-0 plants were treated with 1 μM flg22 and AtPep1 and the expression patterns of the *PP2-B13* and *ACLP1* genes were measured 30 min, 2 h, and 6 h after elicitor treatment. Expression was measured by quantitative reverse transcription (RT)-PCR using gene-specific primers. The X-axis indicates the genes name; the Y-axis indicates the log_2_ scale of the gene expression levels. Each bar represents the fold changes relative to mock samples. Data were normalized using the housekeeping gene *Ubiquitin*. Values were obtained from the means ± SD of three technical replicates and analyzed by Student’s t-test. Two independent experiments were performed with the similar results. *P*-values are indicated *p-value ≤0.05, **p-value ≤0.01, ***p-value ≤0.001.

As can be seen in the volcano plot in Fig 1D, gene expression levels of *At1G56240* and *At1G69900* were strongly induced by flg22. In response to flg22, the expression levels of *At1G56240* and of *At1G69900* were 126-fold and 20-fold induced, respectively (Fig 1D). Similarly, AtPep1 treatment leads to a 120-fold up-regulation of *At1G56240* and a 10-fold up-regulation of *At1G69900* (Fig 1E; the gene expression levels of the genes which are present in Table 1, is presented in the Volcano plot in the S1 Fig). To further monitor the gene expression of *At1G56240* and *At1G69900* upon elicitor perception and to validate the RNA-seq results, we analyzed expression levels by quantitative reverse transcription PCR (qRT-PCR) in leaves of four-week-old Arabidopsis plants at different time points. We confirmed that also at this later developmental stage, expression of *At1G56240* and *At1G69900* was strongly induced (100-fold for *At1G56240* and 12-fold for *At1G69900*) within 30 minutes after flg22 treatment (Fig 4). Two and six hours after elicitor treatment, expression levels of *At1G56240* had returned to pre-treatment levels, while those of *At1G69900* remained only slightly elevated (Fig 4). This expression pattern suggests that both genes might be involved in early defense response. Furthermore, 30 minutes after AtPep1 treatment, the copy number of *At1G56240* mRNA increased very strongly (around a 70-fold change). *At1G69900* expression in response to AtPep1 treatment was up-regulated almost 7-fold, 30 minutes after elicitor treatment. These results show that these genes are strongly activated in the PTI response to both exogenous signal (flg22 treatment) and endogenous signal (AtPep1 treatment). Taken together, it seems that these genes are highly active upon flg22 and AtPep1 treatments in seedlings (based on deep sequencing results; Fig 1D) and also in mature leaves (qRT-PCR results; Fig 4).

According to the Arabidopsis Information Resource [74] and the SIGnAL database (http://signal.salk.edu/), the predicted T-DNA insertion site in SALK_144757.54.50 is located in the second of three exons of *At1G56240* (S2 Fig; panel A); The genomic DNA was extracted from the plants and using the specific primers (S3 Table) in PCR experiment, the T-DNA was detected among insertion lines with the expected size. As can be seen in S2 Fig (panel B), lines 1 and 5 are homozygous mutants. The homozygous mutant line 5 was used for the subsequent study (S2 Fig; panel B). The T-DNA insertion in SALK_68692.47.55 is located in the first of two exons of *At1G69900* (S2 Fig; panel A). As illustrated in S2 Fig (panel C), the T-DNA homozygous mutant line was detected among the T-DNA insertion lines in PCR with the expected size while it was not detected in the wild-type plants. The homozygous mutant line 5 was used for the subsequent study (S2 Fig; panel C). We confirmed that the T-DNA insertion lines were null alleles for *pp2-b13* and *aclp1*, respectively, via reverse-transcription polymerase chain reaction (RT-PCR) (S2 Fig; panel D and E for *pp2-b13* and *aclp1* mutant lines, respectively). *PP2-B13* and *ACLP1* transcripts were not detectable in the respective T-DNA insertion lines (S3 Fig panels A and B); while *ACTIN2* transcript was detected in the control in Col-0 (wild type), *At1G56240* and *At1G69900* (S3 Fig panel C). We therefore, refer to these lines as *pp2-b13* and *aclp1*, respectively. Visual inspection of plant growth did not reveal any obvious phenotypic differences between any of the two insertion lines and wild-type Col-0 with regard to size and shape at the rosette stage (S4 Fig).

### Differential ethylene production in *pp2-b13* and *aclp1* plants, as compared to the wild type Arabidopsis

**Fig 5:**
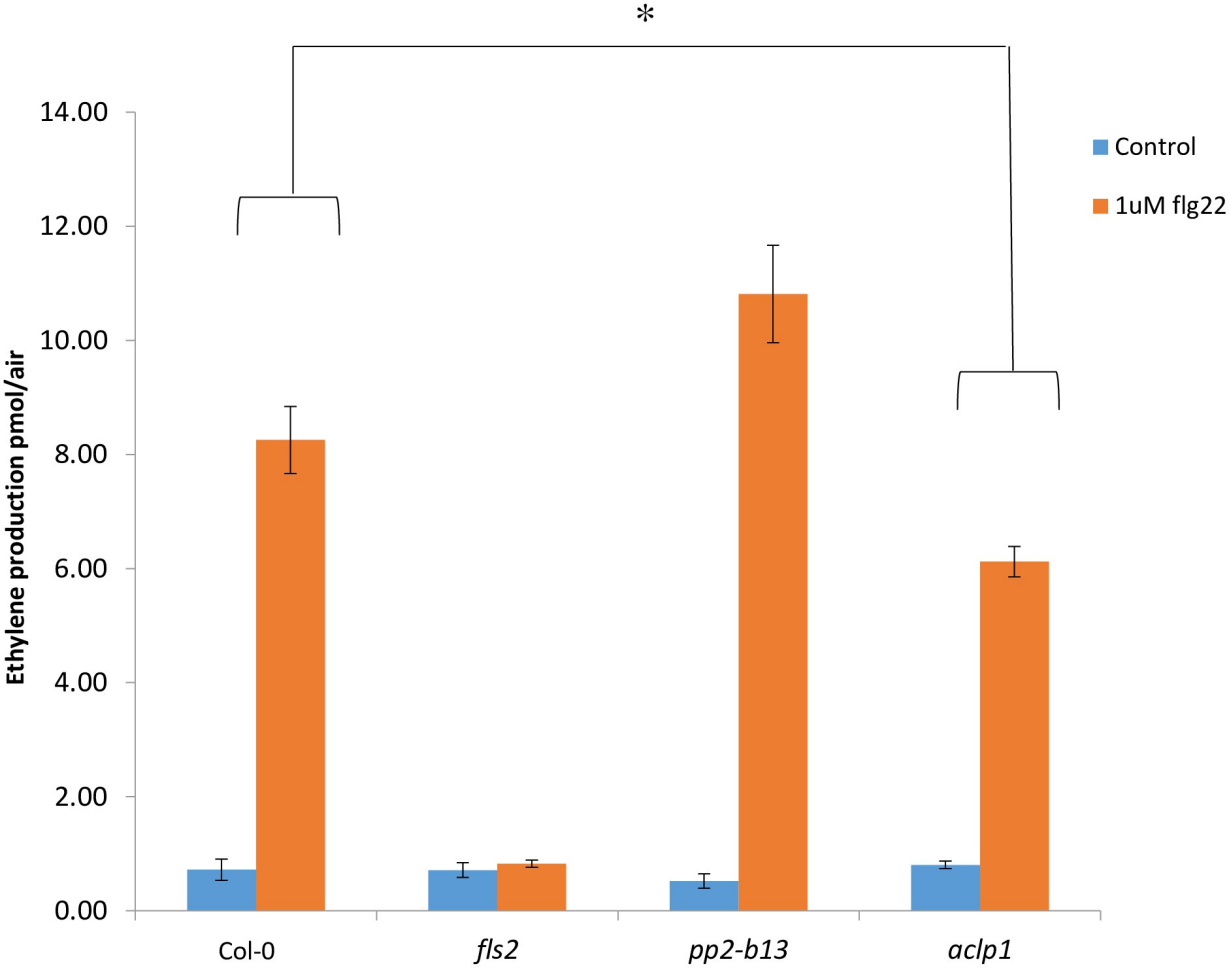
Early PTI responses upon elicitor treatment. Ethylene accumulation after elicitor treatment. Leaf discs of four- to five-week-old plants of wild-type and mutant lines (*pp2-b13*, and *aclp1*) were treated with 1 μM of the flg22 elicitor peptide or without any peptide (control). In all cases, ethylene production was measured three and a half hours after closing the tubes. Ethylene accumulation in *pp2-b13* and *aclp1* mutant lines was compared to the wild-type Arabidopsis. *fls2* mutant line was used as a negative control. Values were obtained from the mean ethylene concentration ± SD of six technical replicates. Similar results were obtained in at least six independent experiments. *T-test* was performed comparing the responses of the control treatment to the elicitor treatments; *P*-values are indicated *p-value ≤0.05. Additional repeats are in the S6 Fig (Panels A-C).

The stress hormone ethylene is typically produced as an early response to an elicitor treatment or to an attack by a plant pathogen [1]. Ethylene measurements, like ROS measurements, are therefore regarded as one of the best reliable techniques to evaluate the plant’s immune response to microbial infection [75]. Therefore, we assessed ethylene (ET) production in response to flg22 treatment in the mutant lines *pp2-b13*, and *aclp1*. We observed that the mutant line *aclp1* displayed a significantly reduced ET production in comparison to wild-type Arabidopsis upon treatment with 1 μM flg22 (*p-*value = 0.0023; Fig 5). In additional ethylene measurement experiments in response to flg22, *aclp1* displayed a statistically significant reduced ethylene production compared to the wild-type plants. As can be seen in the S7 Fig (panels A-C), *aclp1* produced much less ethylene compared to the wild type plants (*p-*value for Panel A = 0.0115; *p-*value for Panel B = 0.0037; *p-*value for Panel C = 0.0023). This suggests that ACLP1 is involved, directly or indirectly, in the enhancement of ET production in response to flg22 perception.

### Differential reactive oxygen species production in *pp2-b13* and *aclp1* plants, as compared to the wild type Arabidopsis

**Fig 6:**
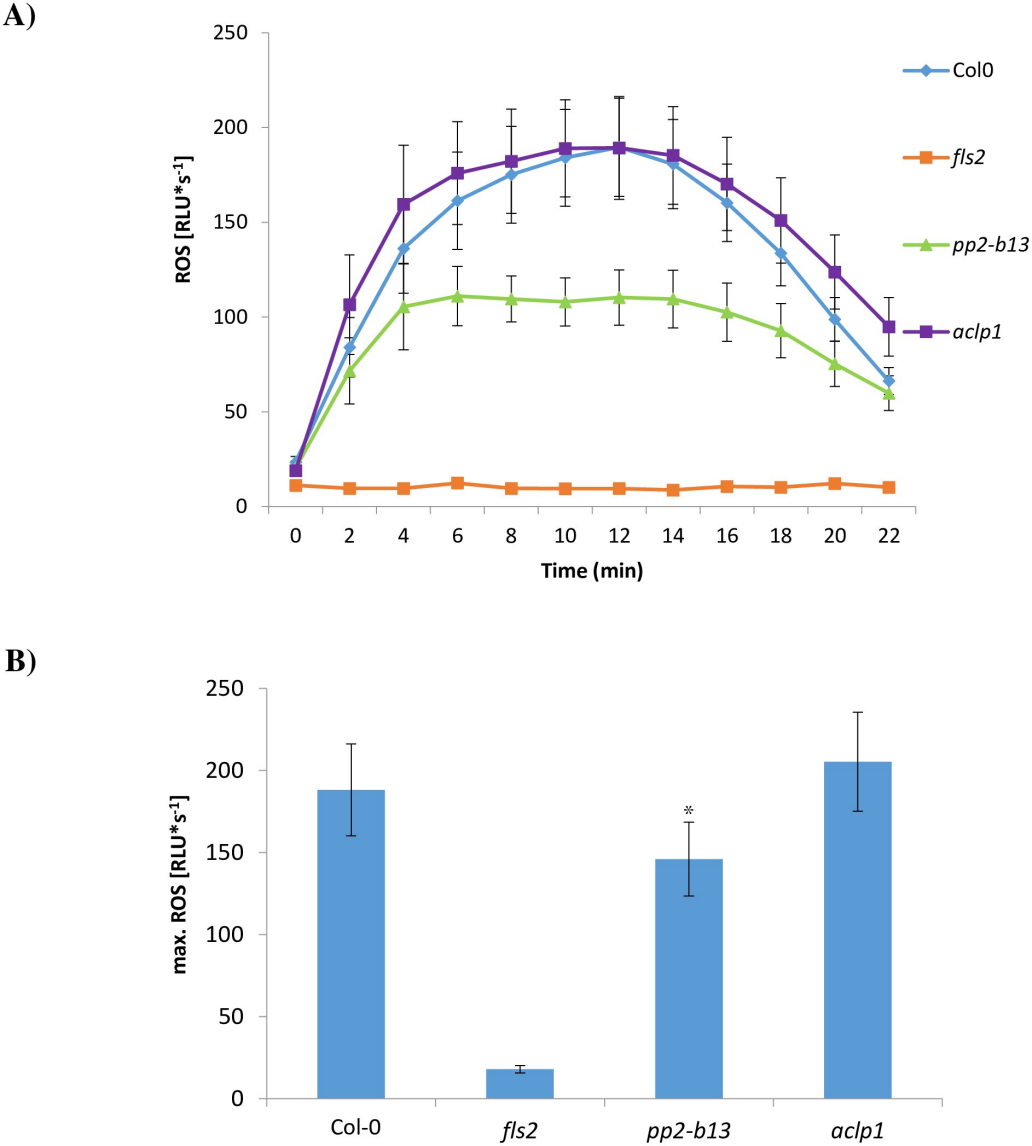
ROS production after treatment with flg22. Leaf discs were treated with 1 μM flg22 or without any peptide (control). (A) indicates ROS production in *pp2-b13* and *aclp1* mutant lines compared to wild-type Arabidopsis; (B) represents maximum ROS production in *pp2-b13* and *aclp1* mutant lines compared to wild-type Arabidopsis. *fls2* mutant line was used as a negative control. Graphs display average of 12 technical replicates. Error bars indicate standard error (SE) of the mean. The experiment was repeated four times with similar results. RLU = relative light units. *T-test* was performed comparing the responses of the control treatment to the elicitor treatments; *P*-values are indicated *p-value ≤0.05.

One of the early responses triggered by MAMPs and DAMPs is the production of apoplastic ROS by the Arabidopsis NADPH-oxidases, RbohD, and RbohF [21]. We observed that in the treated leaf discs upon flg22 perception, *pp2-b13* displayed a lower ROS production compared to wild-type (Fig 6A and 6B), indicating that PP2-B13 might play a role in early PTI by contributing to the oxidative burst in response to the flg22 perception. In contrast, *aclp1*, although exhibiting deficiency in ET production upon flg22 perception, showed robust enhancement of ROS production at levels similar to that of wild-type (Fig 6A and 6B; *p-*value = 0.0468 for *pp2-b13* compared to wild type plants).

### FLS2 receptor abundance in *pp2-b13* and *aclp1* mutants were similar to the wild type Arabidopsis

**Fig 7:**
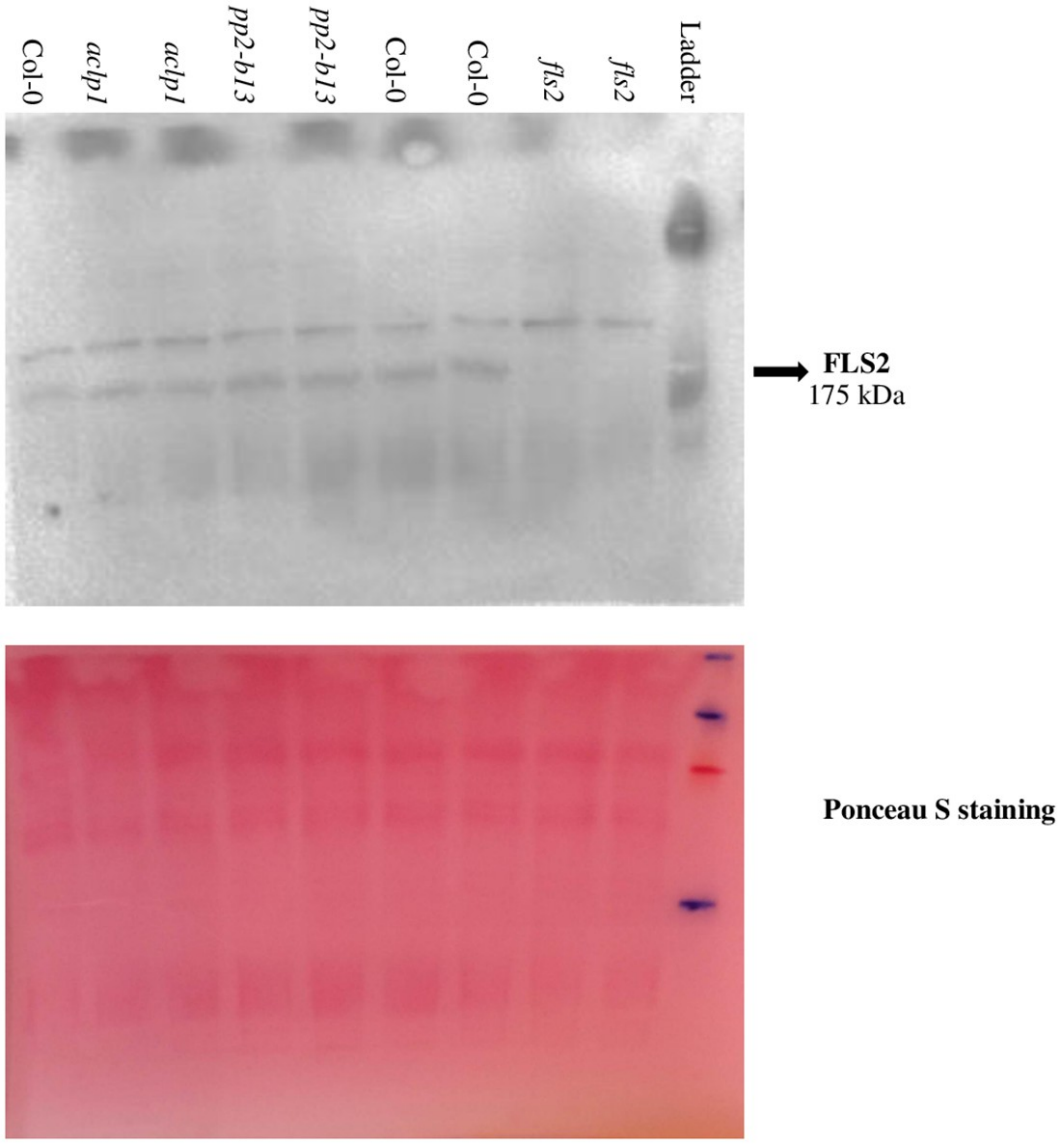
FLS2 protein levels. FLS2 protein levels of the mutant lines *pp2-b13* and *aclp1* as detected by immunoblot using a FLS2-specific antibody. *fls2* mutant plant is used as the negative control. Ponceau S staining was used as the loading control. The original gel image is presented in S7 Fig.

Recent studies showed that there are several proteins that have a role in the abundance of FLS2 in the plasma membrane [76]. Furthermore, proper flg22 sensing and correct signaling require a correct integration of FLS2 in the plasma membrane [76]. In the current study, the *PP2-B13* and *ACLP1* genes were strongly induced upon elicitor treatment, as seen in the RNA-seq and qPCR data. Additionally, both mutant lines were deficient in early PTI responses (ET and ROS measurement). Hence it is conceivable that the products of the *PP2-B13* and *ACLP1* genes affect the abundance of the FLS2 receptor. However, FLS2 analysis via immunoblots showed that both mutant lines had similar levels of FLS2 as the wild-type (Fig 7 and S8 Fig). Thus it appears that the two genes under scrutiny do not have a role in regulating the abundance of the FLS2 receptor.

### Differential salicylic acid levels in *pp2-b13* and *aclp1* plants, as compared to the wild type Arabidopsis

**Fig 8:**
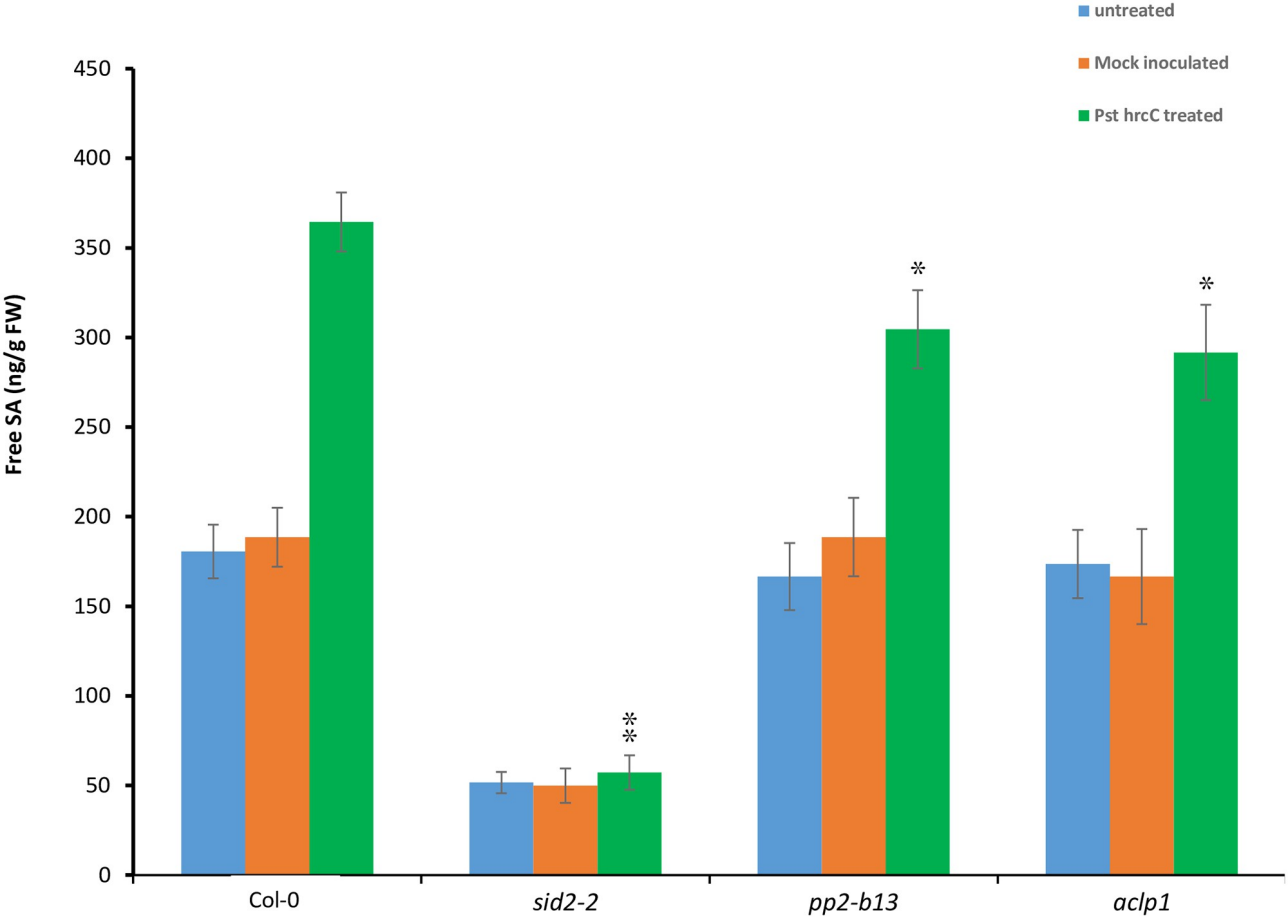
Measurement of free Salicylic acid in *pp2-b13*, *aclp1* and wild-type plants after infiltration with *Psuodomonas syringae* DC3000 *hrcC*. Four- to five-week-old plants of wild-type and mutant lines (*pp2-b13*, and *aclp1*) were infiltrated with *Pst* DC3000 *hrcC* (OD_600_ = 0.0002, in infiltration buffer). Fours leaves for each plants were infiltrated. Treated plants with infiltration buffer regarded as Mock-Control. Six plants were used for each replicates and three technical replicates were taken for each experiments. 48-hour post infiltration the leaves were collected and the free SA levels was measured. *sid2-2* mutant plants, were used as a control. Bars show the mean ± s.e. of three technical replicates. Two independent experiments were performed with the similar results. Statistical analyses were performed using the Students *t*-test. *P*-values are indicated *p-value ≤0.05.

To determine the salicylic acid (SA) level in *pp2-b13* and *aclp1* mutant lines, we measured free SA levels 48 h after *Pst* DC3000 *hrcC* treatments. We observed that in *pp2-b13* and *aclp1* mutant lines, the SA levels were significantly lower than in the wild type plant (*p* = 0.0413 and *p* = 0.0410 for *pp2-b13* and *aclp1*, respectively, Student’s t-test) (Fig 8). This finding, confirmed in two independent experiments, indicated that the protein products of *PP2-B13* and *ACLP1*, are directly or indirectly involved in SA production or SA accumulation.

### Altered expression of the major defense-related marker genes following the infection of *Pseudomonas syringae* DC3000 *hrc*C- in *pp2-b13* and *aclp1* mutant lines

**Fig 9:**
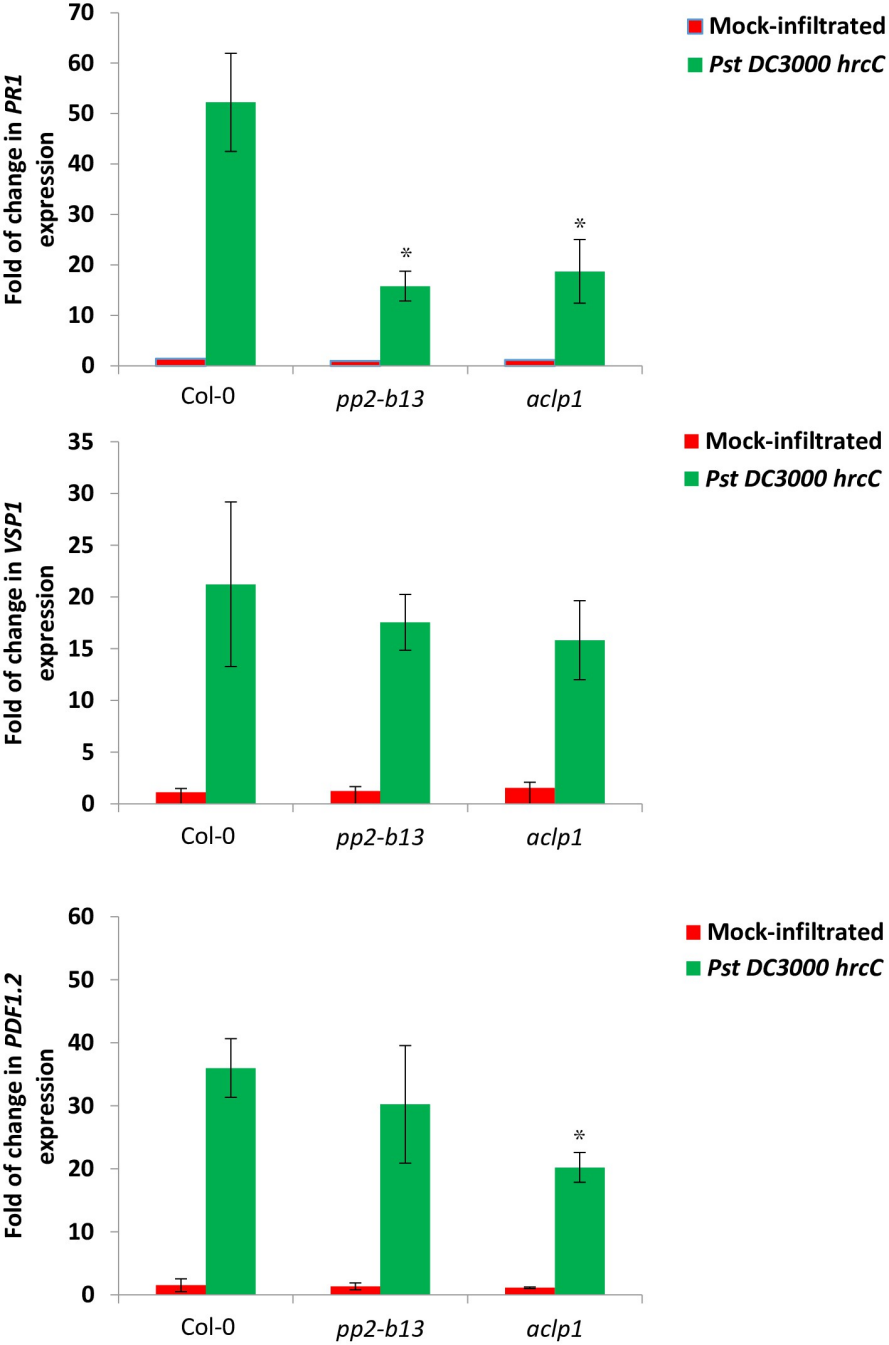
Expression levels of three major defense marker genes in *pp2-b13* and *aclp1* mutant lines compared to the wild-type Arabidopsis (Col-0). Five-week-old plants of wild-type and mutant lines *pp2-b13*, and *aclp1* were pressure infiltrated with *Pst* DC3000 *hrcC* (OD_600_ = 0.0002, in infiltration buffer) and the expression patterns of the genes including *PATHOGENESIS-RELATED GENE1* (*PR1*), *VEGETATIVE STORAGE PROTEIN1* (*VSP1*), and *PLANT DEFENSIN1*.*2* (*PDF1*.*2*) were measured 48 hours after infiltration. Expression was measured by quantitative reverse transcription (RT)-PCR using gene-specific primers. Pressure infiltrated plants with infiltration buffer were regarded as Mock-Control. The X-axis indicates the genes name; the Y-axis indicates the gene expression levels. The relative expression of each marker gene was normalized to that of *ACTIN2* expression. Each bar represents the fold changes relative to mock samples. Values were obtained from the means ± SD of three technical replicates of pooled leaves harvested from six plants for each line. Three independent experiments were performed with the similar results. *P*-values are indicated *p-value ≤0.05, **p-value ≤0.01, ***p-value ≤0.001.

To determine the expression levels of major defense-related marker genes in response to bacterial infection in the mutant lines *pp2-b13* and *aclp1* compared to wild-type Arabidopsis, we examined the five-weeks-old Arabidopsis plants infected with *Pst* DC3000 *hrcC-* and investigated the expression levels of the genes including *PATHOGENESIS-RELATED GENE1* (*PR1; AT2G14610*) the SA-inducible gene, *VEGETATIVE STORAGE PROTEIN1* (*VSP1; AT5G24780*) the JA-inducible gene, and *PLANT DEFENSIN1*.*2* (*PDF1*.*2; AT5G44420*) the JA/Ethylene-inducible gene by quantitative reverse transcription-polymerase chain reaction (qRT-PCR) assay. *PR1*, *VSP1*, and *PDF1*.*2* are well-established defense marker genes that are frequently used to monitor PTI. Their expression levels are affected in response to bacterial infection and they are involved in resistance to microbial pathogens. As can be seen in Fig 9, there were some variations in the levels of gene expression profiles of all tested genes in response to the *Pst* DC3000 *hrcC-* infection in the mutant lines *pp2-b13* and *aclp1* compared to wild-type plants. As shown in Fig 9, 48 post bacterial infiltration, the expression levels of the *PR1* gene in the *pp2-b13* and *aclp1* mutant lines were significantly decreased compared to the wild-type plants (*p*-value = 0.0232 and 0.0448 for *pp2-b13* and *aclp1* mutant lines, respectively). These results suggest that *pp2-b13* directly or indirectly has a role in the SA-related defense. As it is illustrated in Fig 9, the expression levels of the *PDF1*.*2* gene in the *aclp1* mutant line was significantly reduced compared to the wild-type plants (*p*-value = 0.0391). This finding indicates that the ACLP1 has a role in the JA/Ethylene-mediated defense pathway. Furthermore, this finding is consistent with reduced ethylene accumulation results (Fig 5) that displayed a significant reduction in ET production in comparison to the wild-type Arabidopsis. Taken together, our findings suggest that ACLP1 directly or indirectly has a role in ET biosynthesis or ET accumulation.

### Putative protein interactions and protein domains in PP2-B13 and ACLP1

Sequence alignment of PP2-B13 with homologues from other plant species revealed conserved features (S9 Fig). Phylogenetic analysis supported high conservation of PP2-B13-like proteins across different plant species, suggesting a similar function (S10 Fig). *In silico* structural analysis using Raptor X [77] predicted two domains (S11 Fig): an N-terminal F-box domain (residues 4–46; S11 Fig) and a C-terminal PP2 domain (residues 93–280; S10 Fig). Furthermore, to predict PP2-B13 interaction partners, we submitted the PP2-B13 amino acid sequence to the STRING database (version 11.0), which determines hypothetical protein-protein interactions based on computational prediction methods [78]. This returned several major players in innate immunity, specifically PBL1, RLP6, and RLP15, which are important defense proteins, as potential interaction partners (S12 Fig) [79–81]. RLPs are regarded as major players in the immune system in Arabidopsis [79–81]. STRING also predicted interactions of PP2-B13 with major zinc transporter proteins (ZIPs), which have role in biotic and abiotic stress responses [82].

ACLP1 is an actin cross-linking protein of 397 amino acids. Raptor X [77] predicted two Fascin motifs in the N -terminal and C-terminal domains (residues 18–70 and 229–318, respectively; S13 and S14 Figs). The conserved domain database at NCBI (https://www.ncbi.nlm.nih.gov/Structure/cdd/cdd.shtml) also identified two fascin domains in ACLP1. Fascins are a structurally unique and evolutionarily highly conserved group of actin cross-linking proteins. Fascins function in the organization of two major forms of actin-based structures: dynamic, cortical cell protrusions and cytoplasmic microfilament bundles [83–85]. Sequence Logo analysis revealed several conserved regions in the ACLP1 and its homologues (S14 Fig). Furthermore, a phylogenetic analysis supported high conservation of ACLP1-like proteins across different land plant species, suggesting conserved function (S14 Fig).

## Discussion

Although genetic components in response to flg22 have been widely studied, to date little information is available on the comparative differential gene expression analysis in response to exogenous (flg22) and endogenous (AtPep1) elicitors at early time-points. Here, we investigated the commonalities and differences in early immune response activation at the transcriptional level upon flg22 and AtPep1 perception, and whether such a comparative approach could be used to identify novel players in innate immunity. In this research, we could identify numerous genes that were exclusively transcribed in response to flg22 and AtPep1. Our data show that, while the transcription of more genes in response to these two elicitors shared substantial commonalities, with 1,378 genes up-regulated in both conditions, each one also triggered the up-regulation of an elicitor-specific set of genes (256 for flg22 and 328 for AtPep1, respectively; Fig 1B and S6 to S15 Tables). We found that the endogenous peptide AtPep1 can induce the transcription of many genes compared to flg22. We showed that 328 genes were exclusively up-regulated in response to AtPep1 (Fig 1B; S13 Table), and that this effect was stronger for genes suppressed upon elicitor perception, with 107 and 411 flg22- and AtPep1-regulated genes, respectively, compared to only 154 commonly down-regulated genes (Fig 1C; S14 and S15 Tables). This relatively strong response to AtPep1 potentially can be interpreted as underlining the relevance of AtPep1 in reprogramming gene expression during the early immune response. It suggests that the genes that are activated in response to AtPep1 are under the control of highly sensitive regulatory elements. Our findings have been confirmed by a recent investigation by Bjornson et al. (2021), who similarly observed flg22- and AtPep1-specific gene activation. Hence, early immune response seems to be at least partially specific for each of these elicitors. Subsequent investigations are needed to determine the transcription factors and regulatory mechanisms involved in AtPep1 perception. In another recent study that were investigated by Thieffry et al. (2022), they performed an extensive analysis to determine that flg22-induced genes in *A*. *thaliana* often undergo alternative transcription start site (TSS) and hence alternative isoform selection. However, their study did not address the response to an endogenous elicitor such as AtPep1. Given that there is increasing evidence that the different members of the AtPeps family are not redundant and that each AtPep carries out specific functions in innate immunity [13, 16], future studies should investigate the response to these orthologous gene products. Furthermore, we have compared the expression levels of the top 20 candidates’ genes (Table 1) in our study with the expression levels of the genes that were investigated by Bjornson et al. (2021) and Thieffry et al. (2022) Our results are almost the same as what they have found in their RNA-seq analysis (S18 Table). Interestingly, in the comparison with Bjonrson et al. (2021) results, we found that *PP2-B13* is highly up-regulated in response to all treatments that they have done in their experiments. Furthermore, we confirmed the expression levels of these 20 candidate genes that we found in RNA-seq analysis with qPCR methods. As can be seen in Fig 2, the expression levels of these 20 candidate genes are validated by qPCR experiment. This finding validates the robustness of the RNA-seq technique as a powerful tool for global gene analysis investigations.

We were able to identify PP2-B13 and ACLP1 as two novel players in innate immunity. Compared to control treatment, we observed a strong and very rapid but transient induction of *PP2-B13* (>100 fold change) and *ACLP1* (>10 fold change) within 30 min of flg22 elicitor treatment (Figs 2 and 4). Our reverse-genetic study of *pp2-b13*, and *aclp1* revealed that the respective proteins are required for wild-type levels of resistance to *Pst* DC3000 and *Pst* DC3000 *hrcC* (Fig 3). Mutant lines deficient for the *PP2-B13* or *ACLP1* were sensitive to bacterial infection and deficient in early defense responses. In the next step, further investigations are required to determine the function of PP2-B13 and ACLP1 in immune signaling and their interaction with other components in innate immunity. We provide evidence that loss-of-function mutations in *PP2-B13* and *ACLP1* can affect early PTI responses including ET and ROS measurements (Figs 5 and 6). We could show a defect in activation of ET production for *aclp1* plants, attenuated ROS generation in *pp2-b13* plants in response to flg22 treatment, and lower SA levels in *pp2-b13* and *aclp1* after the infection with *Pst* DC3000 *hrcC* (Fig 8). ROS accumulation is regarded as an early PTI event occurring a few minutes after *Pst* inoculation [21]. In addition to that, compared to the wild-type plants, we observed the reduced expression patterns of *PATHOGENESIS-RELATED GENE1* (*PR1*) gene in response to *Pst* DC3000 *hrcC-* in mutant line *pp2-b13* (Fig 9). Furthermore, *PLANT DEFENSIN1*.*2* (*PDF1*.*2*) expression in *aclp1* mutant line compared to the wild-type plants decreased 48 hours post infection with *Pst* DC3000 *hrcC-* (Fig 9). Conclusively, these findings clearly indicate the role of PP2-B13 and ACLP1 in PTI signaling. However, more work is necessary to determine the relationship between these genes in MAMP recognition, bacterial infection, and other signaling cascades in innate immunity.

PP2-B13 [73] is an F-box protein with homology to PP2-B14 [86]. The F-Box domain of PP2-B13 is close to the N-terminus of the protein. PP2-B13 shows the highest similarity in amino acid sequence with AT1G56250, which formerly was reported as an F-box protein [86]. Zhang et al. [87] showed that PP2-B13 and PP2-B14 were highly abundant in phloem upon aphid infection. These genes are located in a cluster of defense-related genes, which supports the hypothesis that they play a role in the defense signaling network. PP2-domain proteins are one of the most abundant and enigmatic proteins in the phloem sap of higher plants [88, 89]. Furthermore, Jia et al. [90], showed that PP2-B11, (another member of the phloem proteins, [73]) is highly induced in response to salt treatment at both transcript and protein levels. They showed that PP2-B11 plays a positive role in response to salt stress.

MAMP perception changes actin arrangements and leads to cytoskeleton remodeling [91, 92]. The cytoskeleton rapidly responds to biotic stresses to support cellular fundamental processes [93–95]. Recently, Henty-Ridilla et al. [96] confirmed that Actin depolymerizing factor 4 (ADF4) has an important role in defense response through cytoskeleton remodeling. They showed that the *adf4* mutant was unresponsive to a bacterial MAMP [97]. Using the STRING database (version 11.0), we predicted many actin-related proteins including ADF4, ACT2, ACT12, PFN2, MRH2, ARK2, and ADF1 as a putative interaction partner for ACLP1 (S16 Fig), further corroborating a potential role for ACLP in defense-related actin reorganization. It is noteworthy that the gene *PP2-A5* is located downstream of the *ACLP1* locus (S17 Fig). The protein product of the *PP2-A5* gene is another member of the Phloem Protein 2 family [98]. The role of PP2-A5 in defense response against insects is already confirmed [98].

Our study reconfirms the importance of chromosome 1 in innate immunity as there are many resistant genes that their protein product has a role in defense including ACLP1, Di19 [99], PP2-A5 [98], PP2-B13, WWR4 [100, 101], and VBF [86], which are the most important defense genes in *Arabidopsis thaliana*, (S18 Fig). Therefore, we suggest that in further studies, this region of chromosome 1, should be evaluated in-depth to identify more genes that have a role in innate immunity. Furthermore, as it was recently shown that ETI potentiates the PTI [36–38], little is known how these two pathways are co-function to provide a more robust immunity. Therefore, for future research, it will be of interest to study the possible role of PP2-B13 and ACLP1 in ETI and evaluate if they have in mutual potentiation in plant immunity.

## Conclusions

In the current study, global gene expression profiling of wild-type Arabidopsis seedlings resulted in the identification of a large number of genes induced by flg22 and *At*Pep1 that had not been detected by the ATH-1 array technology in previous studies. Our results highlight the general usefulness of transcriptomic approaches to identify new players in early defense responses in innate immunity and reveal two new players, PP2-B13 and ACLP1, in this pathway. It should be noted that extending the time points of the elicitor treatment in future studies might help uncover additional players in innate immunity.

## Supporting information

S1 Fig Volcano plot of gene expression in the seedling of *Arabidopsis thaliana* in response to flg22 treatment (A) and AtPep1 treatment (B) in 20 candidate genes. Blue dots correspond to significantly up- and down-regulated DEGs, while non-DEGs are in grey color. Red dots represent the genes selected for subsequent study.
(TIF)

S2 Fig (A) Schematic representation of homozygous T-DNA mutant lines *PP2-B13*, and *ACLP1*. Boxes indicate exons; thin lines indicate introns; bold arrows indicate T-DNA insertions; arrows indicate the direction of the gene LP1, RP1, and LBa1 primers are represented with blue arrows. (B) The PCR-based genotyping results of SALK_144757.54.50 amplify either the intact gene or the T-DNA. The LP and RP refer to *At1G56240* specific primers which are represented in panel A and in the S3 Table. LBa1 refers to the T-DNA left border specific primer which is represented in the S3 Table. The homozygous plants are highlighted in red color. The homozygous plants (lines 1 and 5 for SALK_144757.54.50) produced a T-DNA insertion product, but no wild-type product in the electrophoresis gel results. (C) The PCR-based genotyping results of SALK_68692.47.55 amplify either the intact gene or the T-DNA. The LP and RP refer to *At1G69900* specific primers which are represented in panel A and in S3 Table. LBa1 refers to the T-DNA left border specific primer in the S3 Table. The homozygous plants are highlighted in red color. The homozygous plants (lines 2, 3, 5, and 9 for SALK_68692.47.55) produced a T-DNA insertion product, but no wild-type product in the electrophoresis gel results. (D) RT-PCR results showing the expression of *PP2-B13* in Col-0 (WT), and *pp2-b13* mutant lines. The lower panel shows amplification of *ACTIN2* transcript as an internal control. Numbers 1.1 to 1.4 indicate individual plants for each genotype corresponding to a single line 1 in panel B. The original gel images of the RT-PCR results are presented in S2 Fig. (E) RT-PCR results showing the expression of *ACLP1* in Col-0 (WT) and *aclp1* mutant lines. The lower panel shows amplification of *ACTIN2* transcript as an internal control. Numbers 5.1 to 5.4 indicate individual plants for each genotype corresponding to a single line 5 in panel C. The original gel images of the RT-PCR results are presented in S2 Fig.
(TIF)

S3 Fig RT-PCR results showing transcripts in Col-0 (WT), *pp2-b13* and *aclp1* mutant lines. (A) The PP2-B13 transcript was detected in Col-0 (WT) but not in the *pp2-b13* mutant line; Numbers 1.1 to 1.4 indicate individual plants for each genotype corresponding to a single line 1. (B) The *ACLP1* transcript was detected in Col-0 (WT) but not in the *aclp1* mutant line. Numbers 5.1 to 5.4 indicate individual plants for each genotype corresponding to a single line 5; (C) The amplification of *ACTIN2* transcript as the control in Col-0 (WT), *pp2-b13* and *aclp1*. Numbers 1 to 4 indicate individual plants for each genotype.
(TIF)

S4 Fig Phenotype of five-week-old Arabidopsis plants. Plants were grown under short-day conditions (ten hours light at 21°C and 14 hours dark at 18°C, with 60% humidity).
(TIF)

S5 Fig Phenotype of five-week-old Arabidopsis plants including wild type Arabidopsis (Col-0), *pp2-b13* and *aclp1*, two days after infection with *Pseudomonas syringae* DC3000 *hrcC*. (TIF)

S6 Fig Bacterial susceptibility assay. Leaves of four- to six-week-old Arabidopsis plants were pressure infiltrated with *Pseudomonas syringae* DC3000 *hrc*C- mutant (OD_600_ = 0.0002, in infiltration buffer). *sid2-2* mutant plants, which are deficient in salicylic acid production, were used as a positive control. Black bars indicate bacterial colony from leaf discs of infected leaves just after infiltration (0 day); white bars represent colony-forming units (cfu/cm^2^) 48 h post-inoculation. Bars show the mean ± s.e. of six technical replicates. Six plants were used for each line. Similar results were observed in four independent experiments. Asterisks indicate a significant difference (*p-value ≤0.05, **p-value ≤0.01) from the wild-type plants as determined by Student’s *t-test*.
(TIF)

S7 Fig Ethylene accumulation after elicitor treatment. Leaf discs of four to five weeks old of the mutant lines (*pp2-b13*, *aclp1*) and also wild-type plants were treated with 1 μM of the flg22 elicitor peptide or without any peptide (control). f*ls2* mutant line was used as a negative control. In all cases, ethylene production was measured three and half hours after closing tubes. Panel (A), (B) and (C); indicate ethylene accumulation in *pp2-b13* and *aclp1* mutant lines compared to the wild type Arabidopsis. Values were obtained from the mean ethylene concentration ± SD of six technical replicates. Similar results were obtained in at least six independent experiments. T‐test was performed comparing the responses of the control treatment to the elicitor treatments; *P*-values are indicated *p-value ≤0.05.
(TIF)

S8 Fig The original gel images of the immunoblot assay and ponceau S staining results. FLS2 protein levels of the Col-0, *aclp1* and *pp2-b13* was detected by immunoblot using a FLS2-specific antibody. *fls2* mutant plant is used as negative control. The original gel image is presented in S7 Fig. Ponceau S staining was used as loading control.
(TIF)

S9 Fig Different sequence conservation profiles in the PP2-B13 and its homologues in different plant species. Conservation plots were constructed using WEBLOGO. The y-axis represents the probability score. Y = 4 corresponds to 100% conservation. The predicted domains are highlighted in red boxes.
(TIF)

S10 Fig Phylogenetic analysis from the sequences of PP2-B13 protein in *Arabidopsis thaliana* and 15 representative land plants. The species indicated are *Ricinus communis*, *Populus trichocarpa*, *Vitis vinifera*, *Beta vulgaris*, *Coffea canephora*, *Nicotiana tomentosiformis*, *Morus notabilis*, *Citrus sinensis*, *Citrus clementine*, *Tarenaya hassleriana*, *Arabidopsis thaliana*, *Capsella rubella*, *Arabis alpina*, *Eutrema salsugineum*, *Brassica rapa* and *Brassica napus*. PP2-B13 protein in *Arabidopsis thaliana* was labelled. Sequences for comparisons were obtained from GenBank. The accession numbers and protein names (if available) are given. Analysis was done by maximum likelihood method implemented in MEGA6 (Molecular Evolutionary Genetics Analysis) version 6.0.
(TIF)

S11 Fig Structure of PP2-B13 protein determined by Raptor X (Källberg et al. [77]). (TIF)

S12 Fig The protein-protein interaction (PPI) network of the PP2-B13 protein in *Arabidopsis thaliana* based on STRING 11.0. Analysis with a confidence threshold score of 0.4 (Szklarczyk et al. [78]). Line colors indicate the type of interaction used for the predicted associations: gene fusion (red), gene neighborhood (green), co-occurrence across genomes (blue), co-expression (black), experimental (purple), text mining (light green); association in curated databases (light blue). Line thickness represents the strength of data support. Proteins that have a known function in the immune response are marked with dotted lines.
(TIF)

S13 Fig Structure of ACLP1 protein determined by Raptor X (Källberg et al. [77]). (TIF)

S14 Fig Different sequence conservation profiles in the ACLP1 and its homologous in different plant species. Conservation plots were constructed using WEBLOGO. The y-axis represents the probability score. Y = 4 corresponds to 100% conservation. The predicted domains are highlighted in red boxes.
(TIF)

S15 Fig Phylogenetic analysis from the sequences of ACLP1 protein in *Arabidopsis thaliana* and 14 representative land plants. The species indicated are *Capsella rubella*, *Camelina sativa*, *Arabidopsis thaliana*, *Arabidopsis lyrata*, *Brassica napus*, *Raphanus sativus*, *Brassica oleracea*, *Arabis alpina*, *Arabis nemorensis*, *Tarenaya hassleriana*, *Carica papaya*, *Pistacia vera*, *Manihut esculenta* and *Hevea brasiliensis*. ACLP1 protein in *Arabidopsis thaliana* was labeled. Sequences for comparisons were obtained from GenBank. The accession numbers and protein names (if available) are given. Analysis was done by the maximum likelihood method implemented in MEGA6 (Molecular Evolutionary Genetics Analysis) version 6.0.
(TIF)

S16 Fig The protein-protein interaction (PPI) network of ACLP1 proteins in *Arabidopsis thaliana* based on STRING 11.0. Analysis with a confidence threshold score of 0.4 (Szklarczyk et al. [78]). Line colors indicate the type of interaction used for the predicted associations: gene fusion (red), gene neighborhood (green), co-occurrence across genomes (blue), co-expression (black), experimental (purple), text mining (light green); association in curated databases (light blue). Line thickness represents the strength of data support. Proteins that have a known function in the immune response are marked with dotted lines.
(TIF)

S17 Fig Integrative genomics viewer (IGV) visualization of alignments and coverage of the Illumina reads at the *ACLP1* locus. (A) Overlaid depth graphs. (B) Zoomed in view of A. In the graph *ACLP1* and *PP2-A5* genes are illustrated.
(TIF)

S18 Fig Integrative genomics viewer (IGV) visualization of alignments and coverage of the Illumina reads at the *PP2-B13* locus. Coverage depth graphs represent transcript abundance. (A) Overlaid depth graphs. (B) Zoomed in view of A. In the graph *PP2-B13*, *VBF* and *WRR4* genes are illustrated.
(TIF)

S1 Table Summary of Illumina sequencing data and mapped reads of Arabidopsis thaliana wild-type (Col-0) under BSA, flg22, and AtPep1 treatments. (XLS)

S2 Table List of 85 selected genes for subsequent study. (XLS)

S3 Table List of the oligonucleotide primers which were used in this study. (XLS)

S4 Table List of all genes in response to flg22 treatment compared to control. (XLS)

S5 Table List of all genes in response to AtPep1 treatment compared to control. (XLS)

S6 Table List of top up-regulated DEGs in response to flg22 treatment compared to control. (XLS)

S7 Table List of top down-regulated DEGs in response to flg22 treatment compared to control. (XLS)

S8 Table List of top up-regulated DEGs in response to AtPep1 treatment compared to control. (XLS)

S9 Table List of top down-regulated DEGs in response to AtPep1 treatment compared to control. (XLS)

S10 Table List of up-regulated DEGs in response to flg22 treatment compared to the AtPep1 treatment. (XLS)

S11 Table List of down-regulated DEGs in response to flg22 treatment compared to the AtPep1 treatment. (XLS)

S12 Table List of DEGs exclusively up-regulated in response to flg22 treatment compared to control. (XLS)

S13 Table List of DEGs exclusively up-regulated in response to AtPep1 treatment compared to control. (XLSX)

S14 Table List of DEGs exclusively down-regulated in response to flg22 treatment compared to control. (XLSX)

S15 Table List of DEGs exclusively down-regulated in response to AtPep1 treatment compared to control. (XLS)

S16 Table List of the up-regulated DEGs with fold change cutoff (adjusted *p*-value < 0.05 and a minimum two-fold change) in response to flg22 treatment compared to the control that are present in both RNA-seq experiment analysis and ATH1 Affymetirx GeneChip. (XLS)

S17 Table List of the up-regulated DEGs with fold change cutoff (adjusted *p*-value < 0.05 and a minimum two-fold change) in response to flg22 treatment compared to control that are exclusively present in RNA-seq experiment analysis. (XLS)

S18 Table Comparison of the 20 top up-regulated genes (RNA-seq analysis) with the Bjornson et al. (2021) and Thieffry et al. (2022) research work. (XLS)

S1 Raw images (DOCX)

## Abbreviations

DAMPs: Damage-associated molecular patterns
DEGs: differentially expressed genes
MAMPs: microbe-associated molecular patterns
NGS: next generation sequencing
PEPR: *At*Pep-receptor
PP2: phloem protein 2
PRR: pattern-recognition receptor
PTI: pattern-triggered immunity
ROS: reactive oxygen species
