# Supplementary figures and images for: Comparative RNA-seq analysis of Arabidopsis thaliana response to AtPep1 and flg22, reveals the identification of PP2-B13 and ACLP1 as new members in pattern-triggered immunity
Source: PLoS One. 2024 Jun 4;19(6):e0297124. doi: 10.1371/journal.pone.0297124 (PMC11149889; doi:10.1371/journal.pone.0297124)

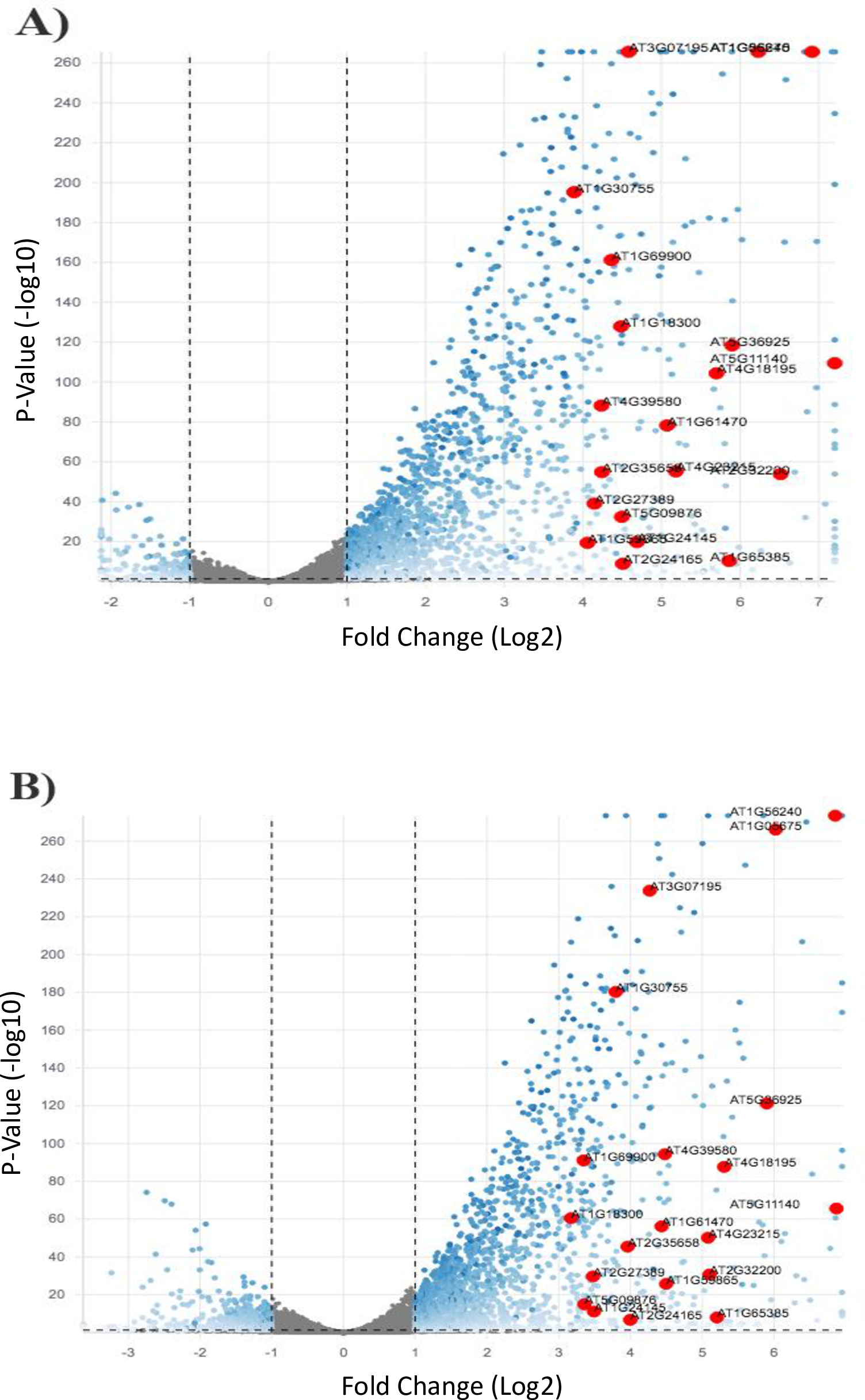

Supplement: S1 Fig — Blue dots correspond to significantly up- and down-regulated DEGs, while non-DEGs are in grey color. Red dots represent the genes selected for subsequent study. (TIF) [file pone.0297124.s001.tif]

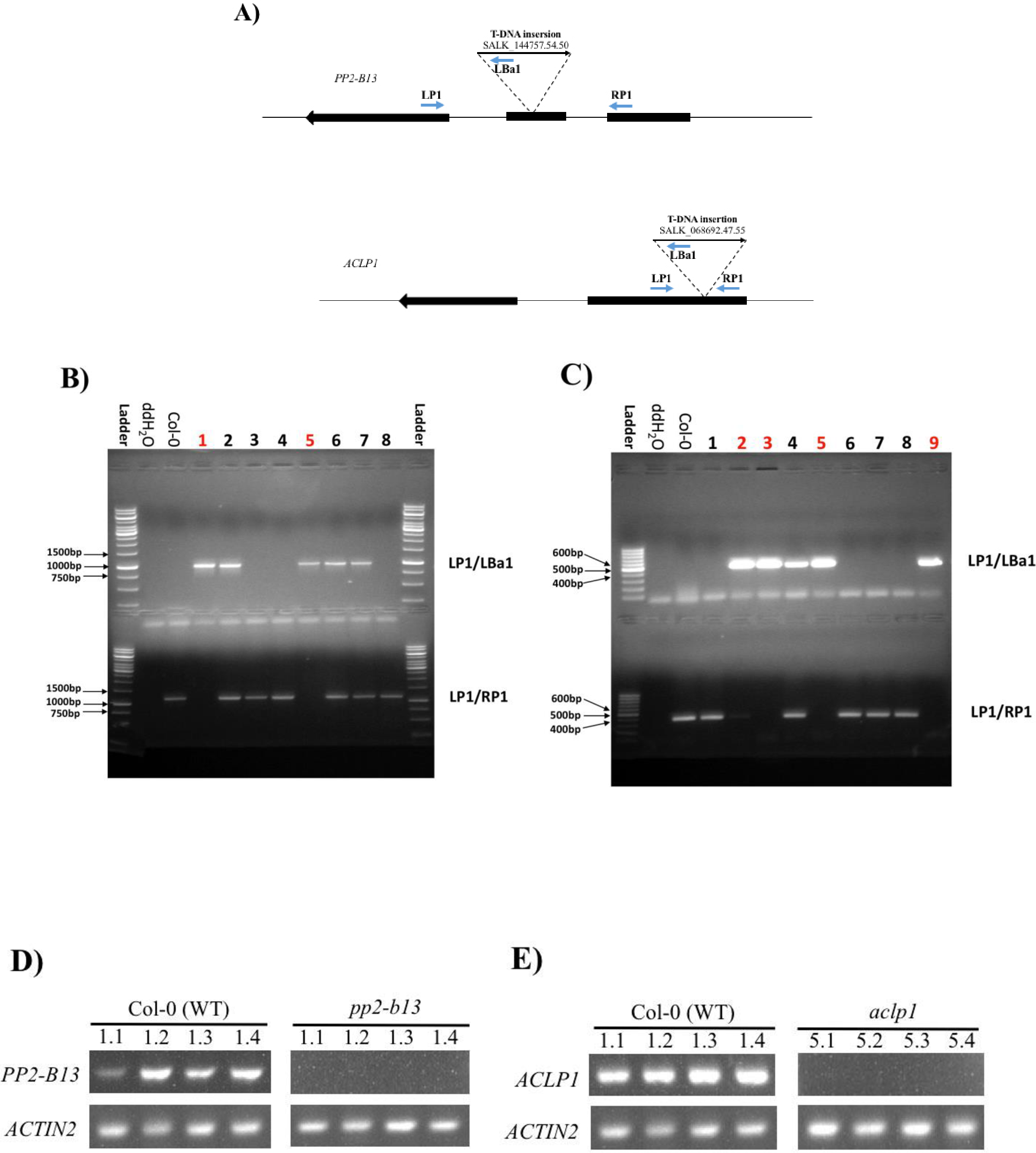

Supplement: S2 Fig — (A) Schematic representation of homozygous T-DNA mutant lines PP2-B13, and ACLP1. Boxes indicate exons; thin lines indicate introns; bold arrows indicate T-DNA insertions; arrows indicate the direction of the gene LP1, RP1, and LBa1 primers are represented with blue arrows. (B) The PCR-based genotyping results of SALK_144757.54.50 amplify either the intact gene or the T-DNA. The LP and RP refer to At1G56240 specific primers which are represented in panel A and in the S3 Table. LBa1 refers to the T-DNA left border specific primer which is represented in the S3 Table. The homozygous plants are highlighted in red color. The homozygous plants (lines 1 and 5 for SALK_144757.54.50) produced a T-DNA insertion product, but no wild-type product in the electrophoresis gel results. (C) The PCR-based genotyping results of SALK_68692.47.55 amplify either the intact gene or the T-DNA. The LP and RP refer to At1G69900 specific primers which are represented in panel A and in S3 Table. LBa1 refers to the T-DNA left border specific primer in the S3 Table. The homozygous plants are highlighted in red color. The homozygous plants (lines 2, 3, 5, and 9 for SALK_68692.47.55) produced a T-DNA insertion product, but no wild-type product in the electrophoresis gel results. (D) RT-PCR results showing the expression of PP2-B13 in Col-0 (WT), and pp2-b13 mutant lines. The lower panel shows amplification of ACTIN2 transcript as an internal control. Numbers 1.1 to 1.4 indicate individual plants for each genotype corresponding to a single line 1 in panel B. The original gel images of the RT-PCR results are presented in S2 Fig. (E) RT-PCR results showing the expression of ACLP1 in Col-0 (WT) and aclp1 mutant lines. The lower panel shows amplification of ACTIN2 transcript as an internal control. Numbers 5.1 to 5.4 indicate individual plants for each genotype corresponding to a single line 5 in panel C. The original gel images of the RT-PCR results are presented in S2 Fig. (TIF) [file pone.0297124.s002.tif]

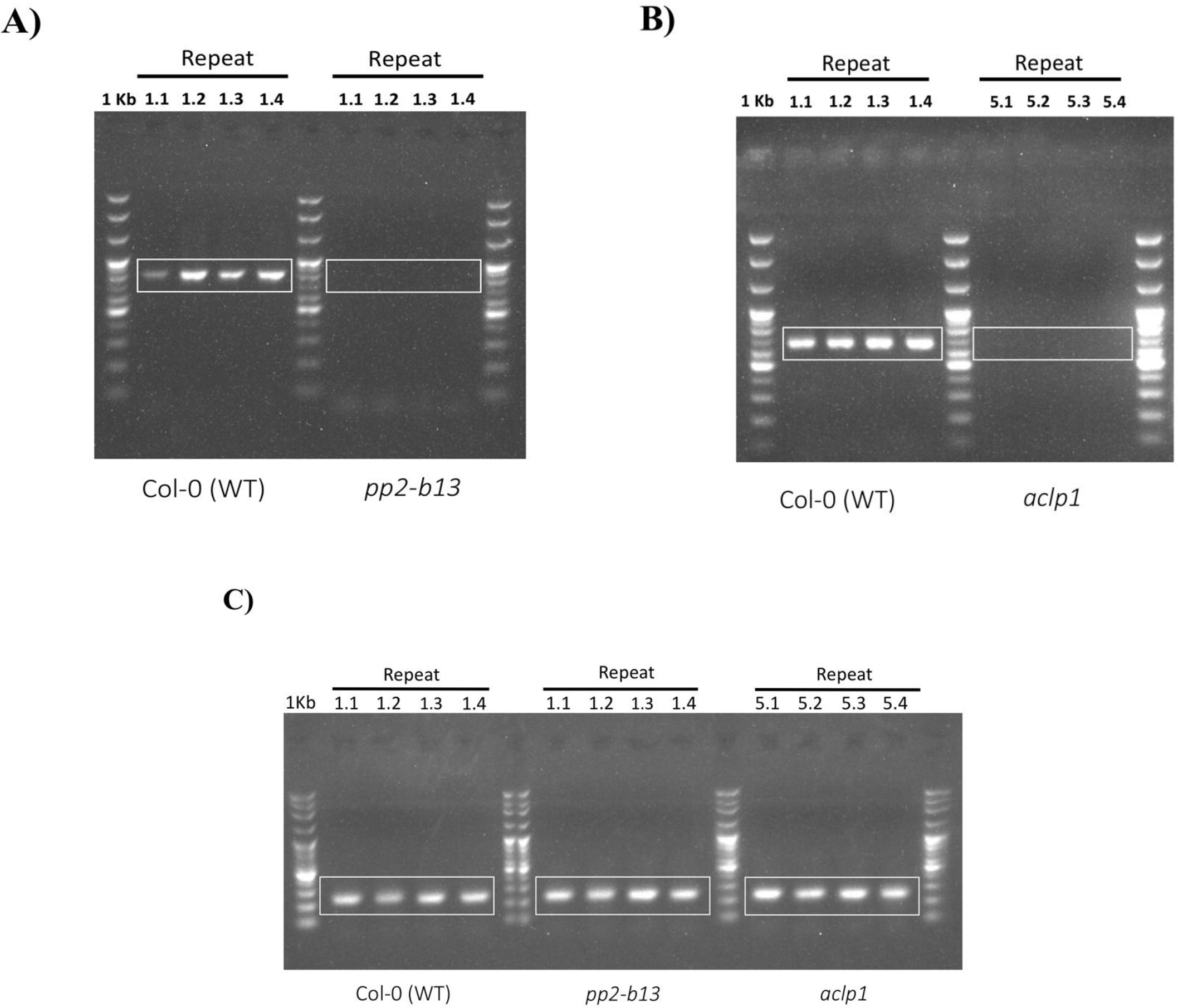

Supplement: S3 Fig — (A) The PP2-B13 transcript was detected in Col-0 (WT) but not in the pp2-b13 mutant line; Numbers 1.1 to 1.4 indicate individual plants for each genotype corresponding to a single line 1. (B) The ACLP1 transcript was detected in Col-0 (WT) but not in the aclp1 mutant line. Numbers 5.1 to 5.4 indicate individual plants for each genotype corresponding to a single line 5; (C) The amplification of ACTIN2 transcript as the control in Col-0 (WT), pp2-b13 and aclp1. Numbers 1 to 4 indicate individual plants for each genotype. (TIF) [file pone.0297124.s003.tif]

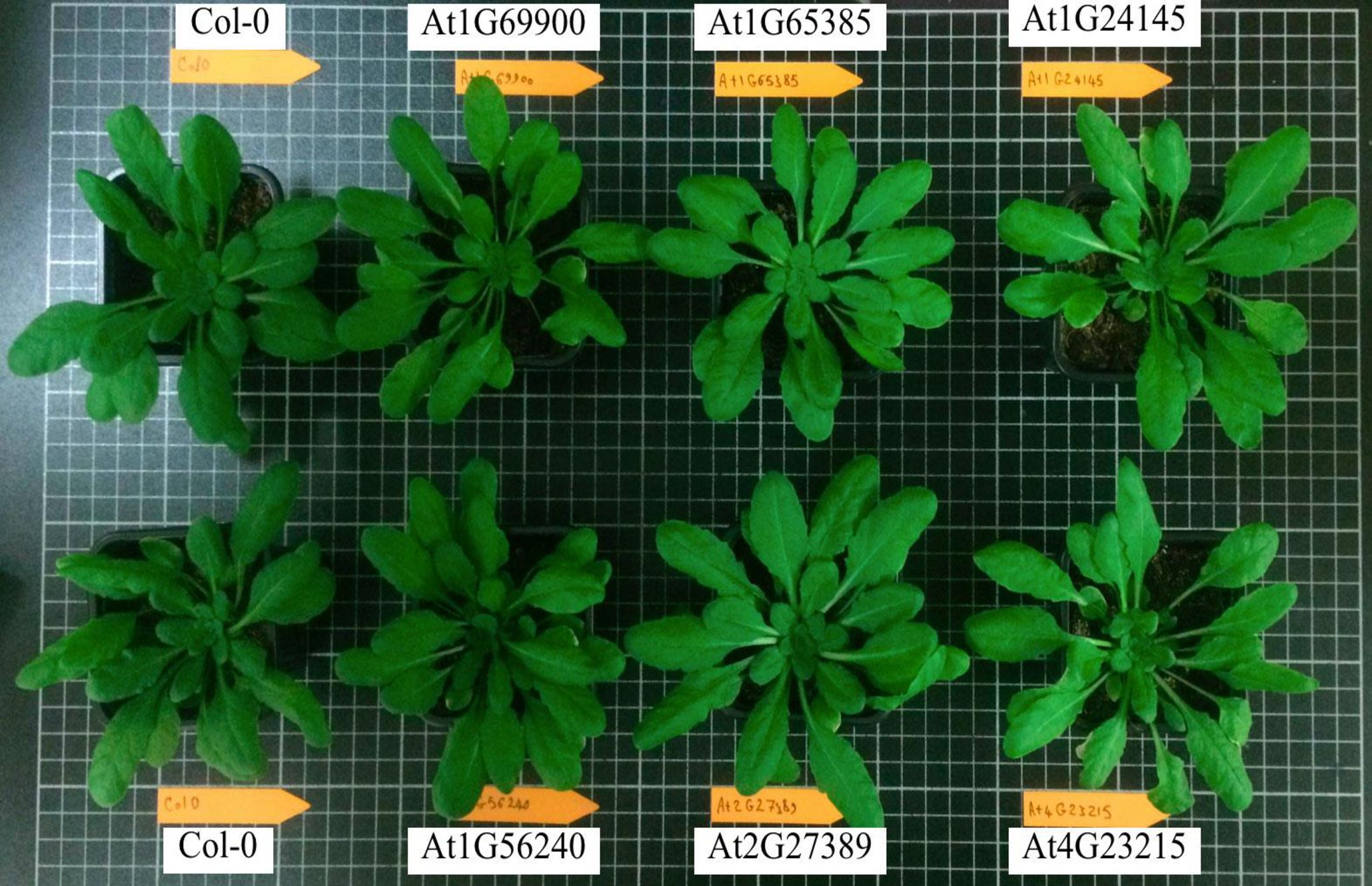

Supplement: S4 Fig — Plants were grown under short-day conditions (ten hours light at 21°C and 14 hours dark at 18°C, with 60% humidity). (TIF) [file pone.0297124.s004.tif]

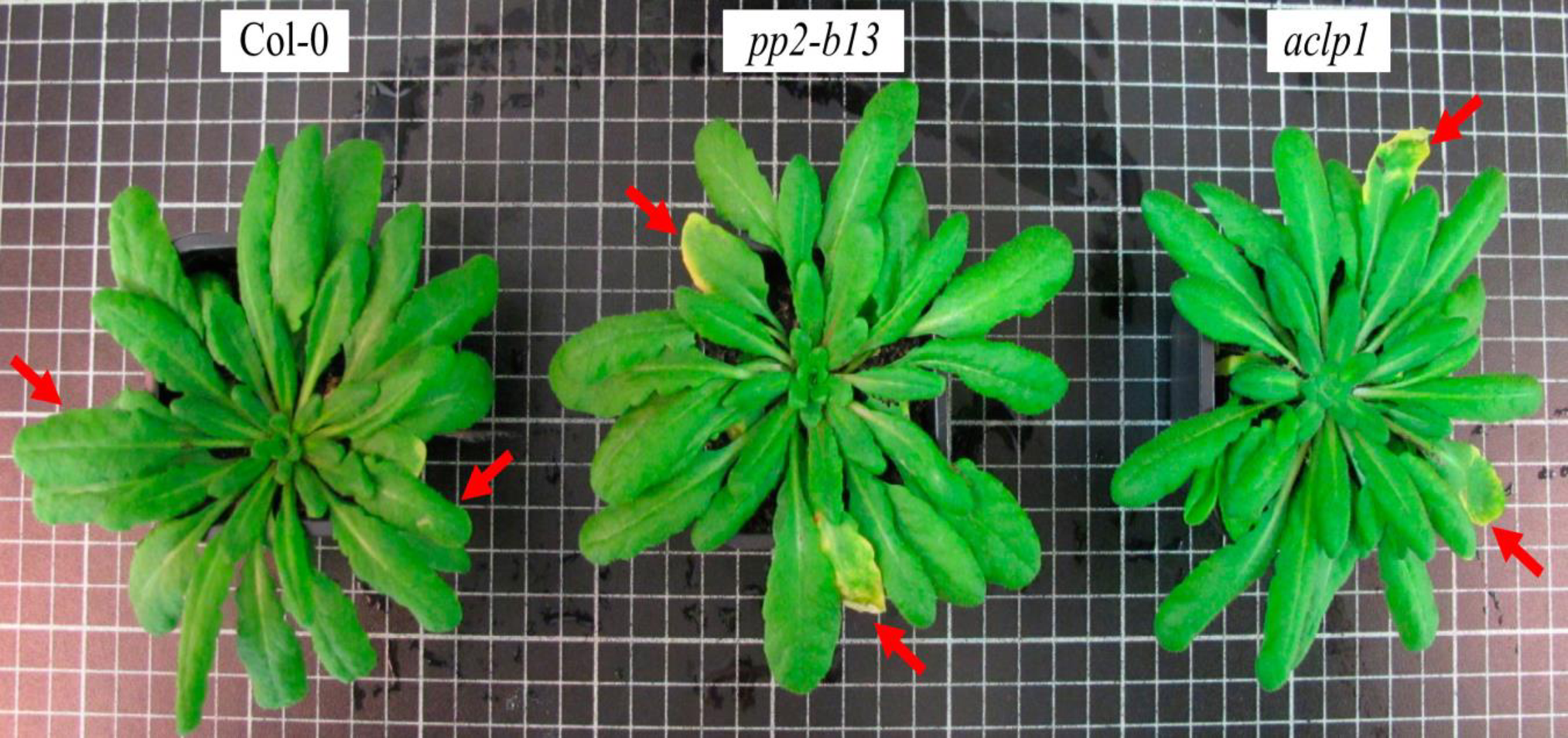

Supplement: S5 Fig — (TIF) [file pone.0297124.s005.tif]

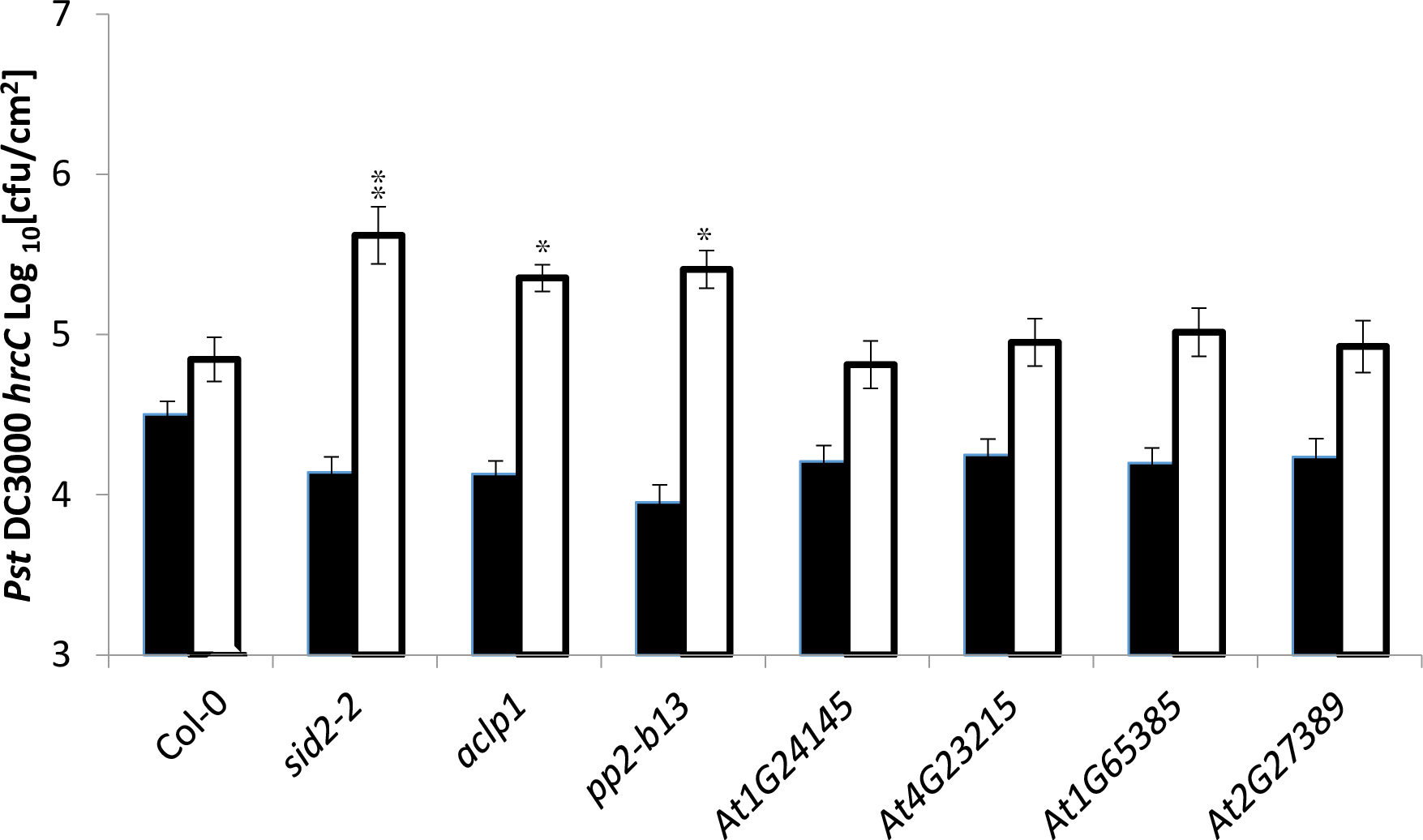

Supplement: S6 Fig — Leaves of four- to six-week-old Arabidopsis plants were pressure infiltrated with Pseudomonas syringae DC3000 hrcC- mutant (OD600 = 0.0002, in infiltration buffer). sid2-2 mutant plants, which are deficient in salicylic acid production, were used as a positive control. Black bars indicate bacterial colony from leaf discs of infected leaves just after infiltration (0 day); white bars represent colony-forming units (cfu/cm2) 48 h post-inoculation. Bars show the mean ± s.e. of six technical replicates. Six plants were used for each line. Similar results were observed in four independent experiments. Asterisks indicate a significant difference (*p-value ≤0.05, **p-value ≤0.01) from the wild-type plants as determined by Student’s t-test. (TIF) [file pone.0297124.s006.tif]

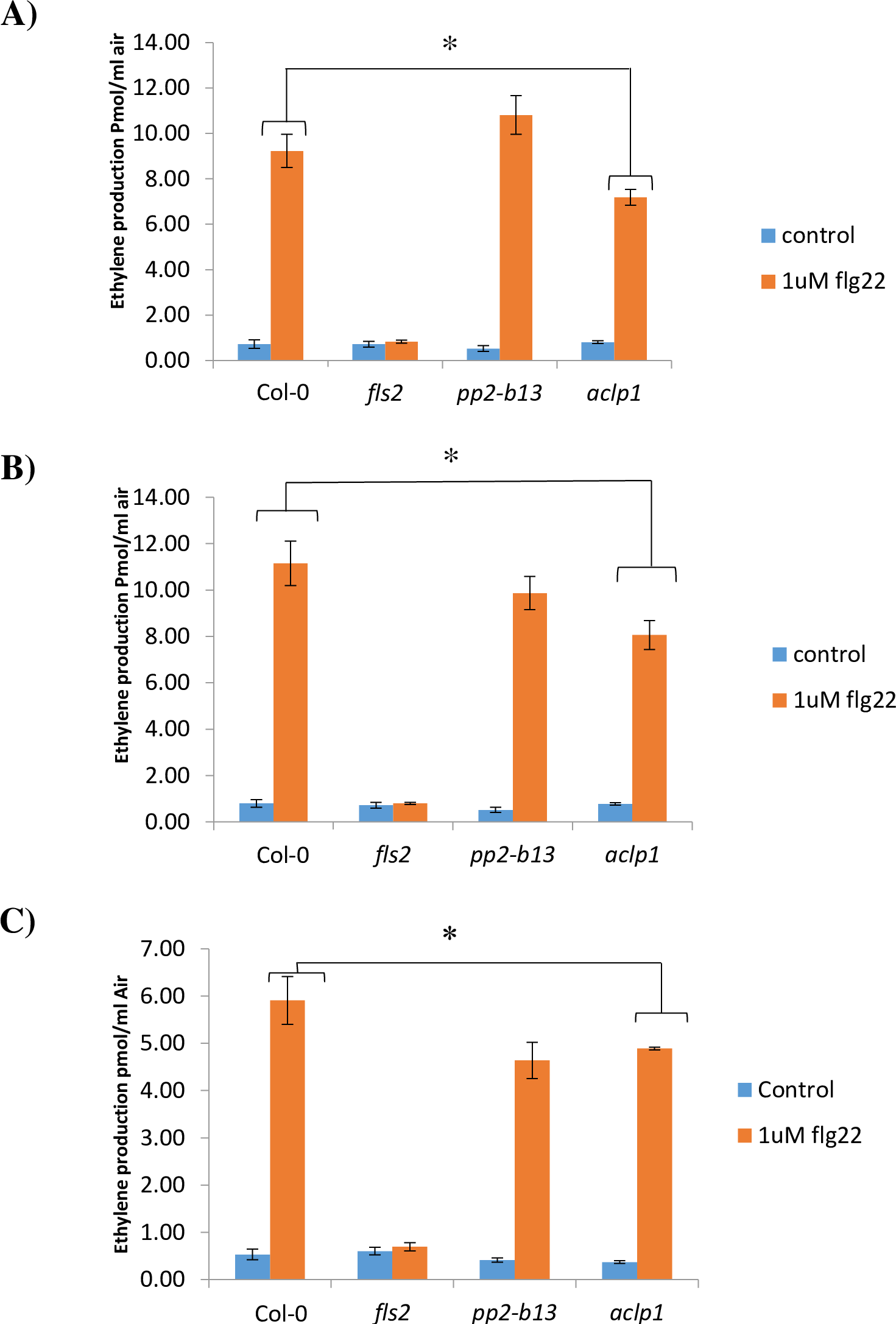

Supplement: S7 Fig — Leaf discs of four to five weeks old of the mutant lines (pp2-b13, aclp1) and also wild-type plants were treated with 1 μM of the flg22 elicitor peptide or without any peptide (control). fls2 mutant line was used as a negative control. In all cases, ethylene production was measured three and half hours after closing tubes. Panel (A), (B) and (C); indicate ethylene accumulation in pp2-b13 and aclp1 mutant lines compared to the wild type Arabidopsis. Values were obtained from the mean ethylene concentration ± SD of six technical replicates. Similar results were obtained in at least six independent experiments. T‐test was performed comparing the responses of the control treatment to the elicitor treatments; P-values are indicated *p-value ≤0.05. (TIF) [file pone.0297124.s007.tif]

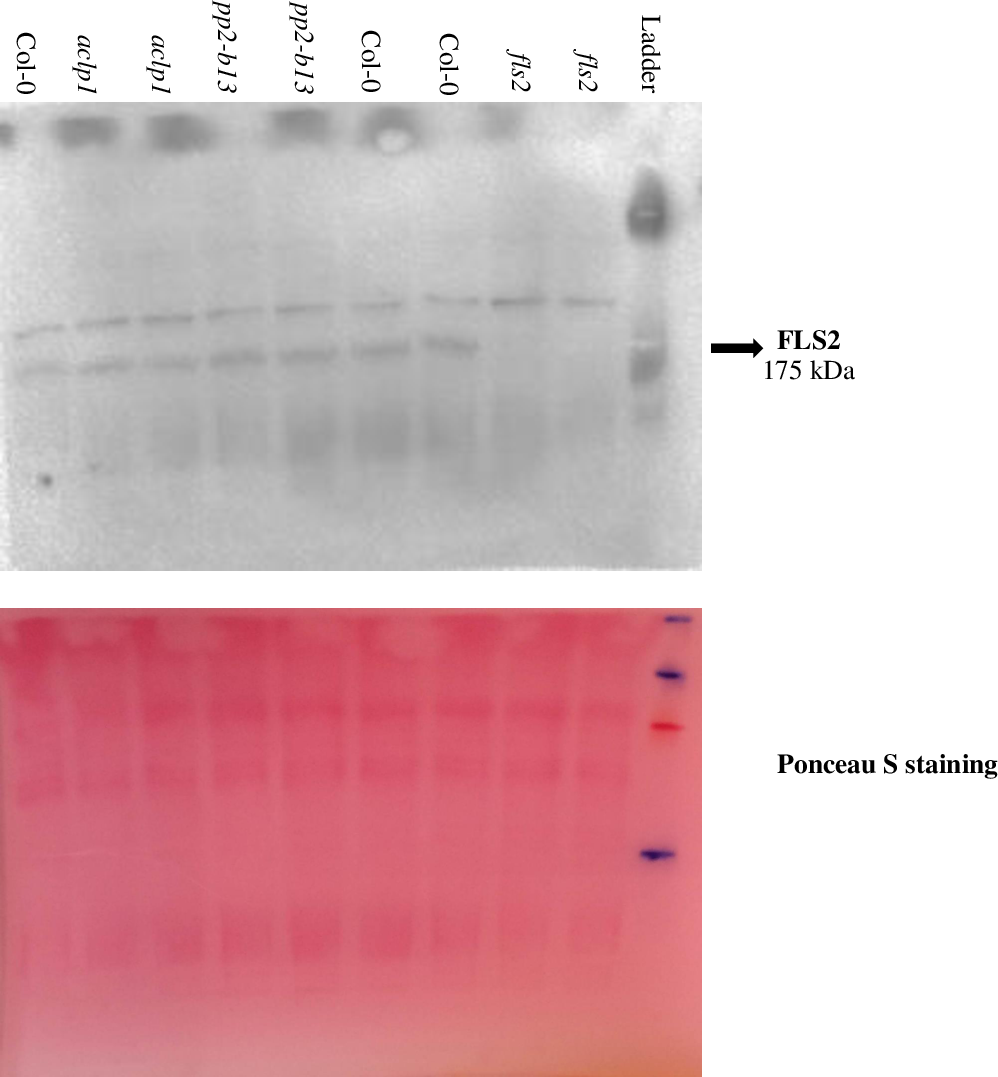

Supplement: S8 Fig — FLS2 protein levels of the Col-0, aclp1 and pp2-b13 was detected by immunoblot using a FLS2-specific antibody. fls2 mutant plant is used as negative control. The original gel image is presented in S7 Fig. Ponceau S staining was used as loading control. (TIF) [file pone.0297124.s008.tif]

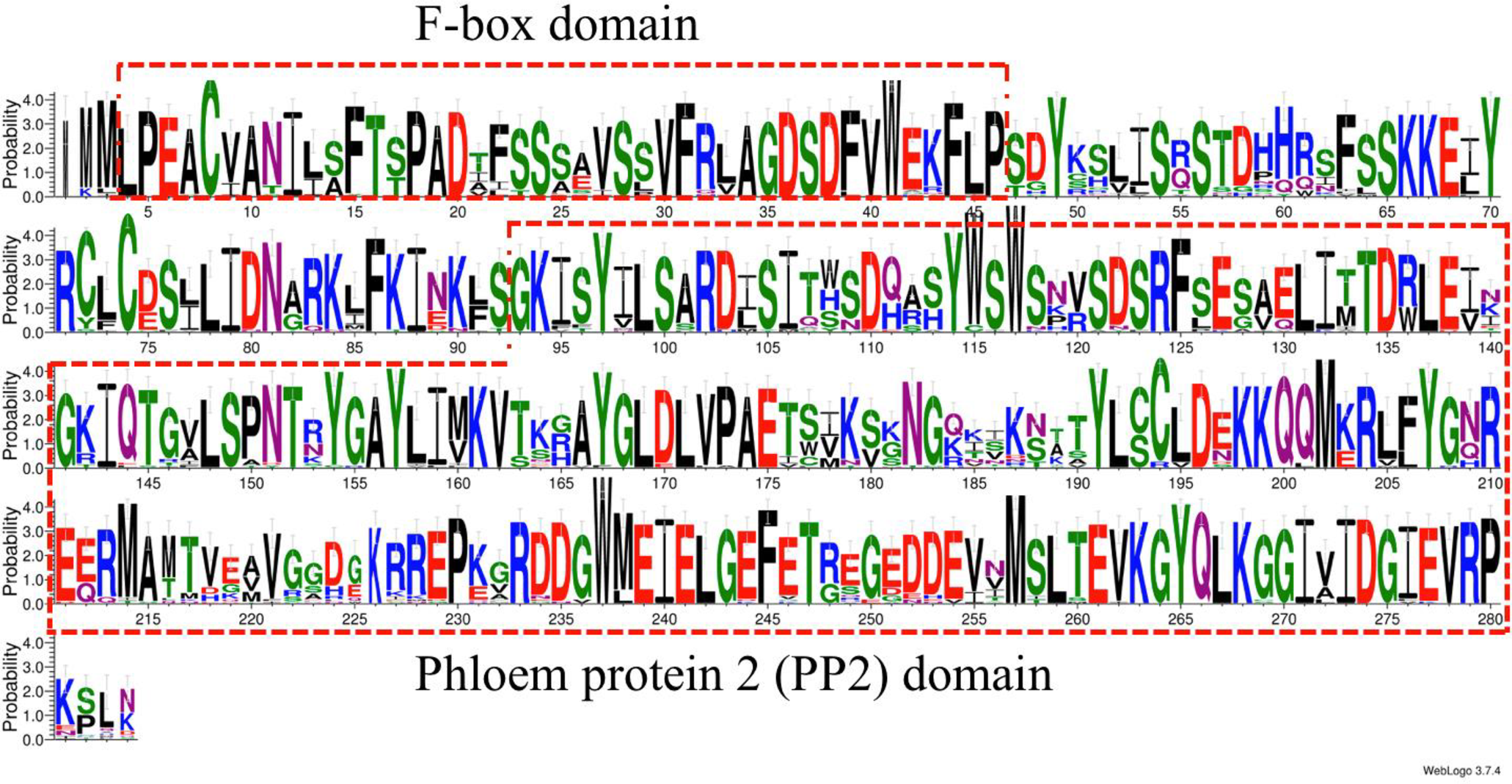

Supplement: S9 Fig — Conservation plots were constructed using WEBLOGO. The y-axis represents the probability score. Y = 4 corresponds to 100% conservation. The predicted domains are highlighted in red boxes. (TIF) [file pone.0297124.s009.tif]

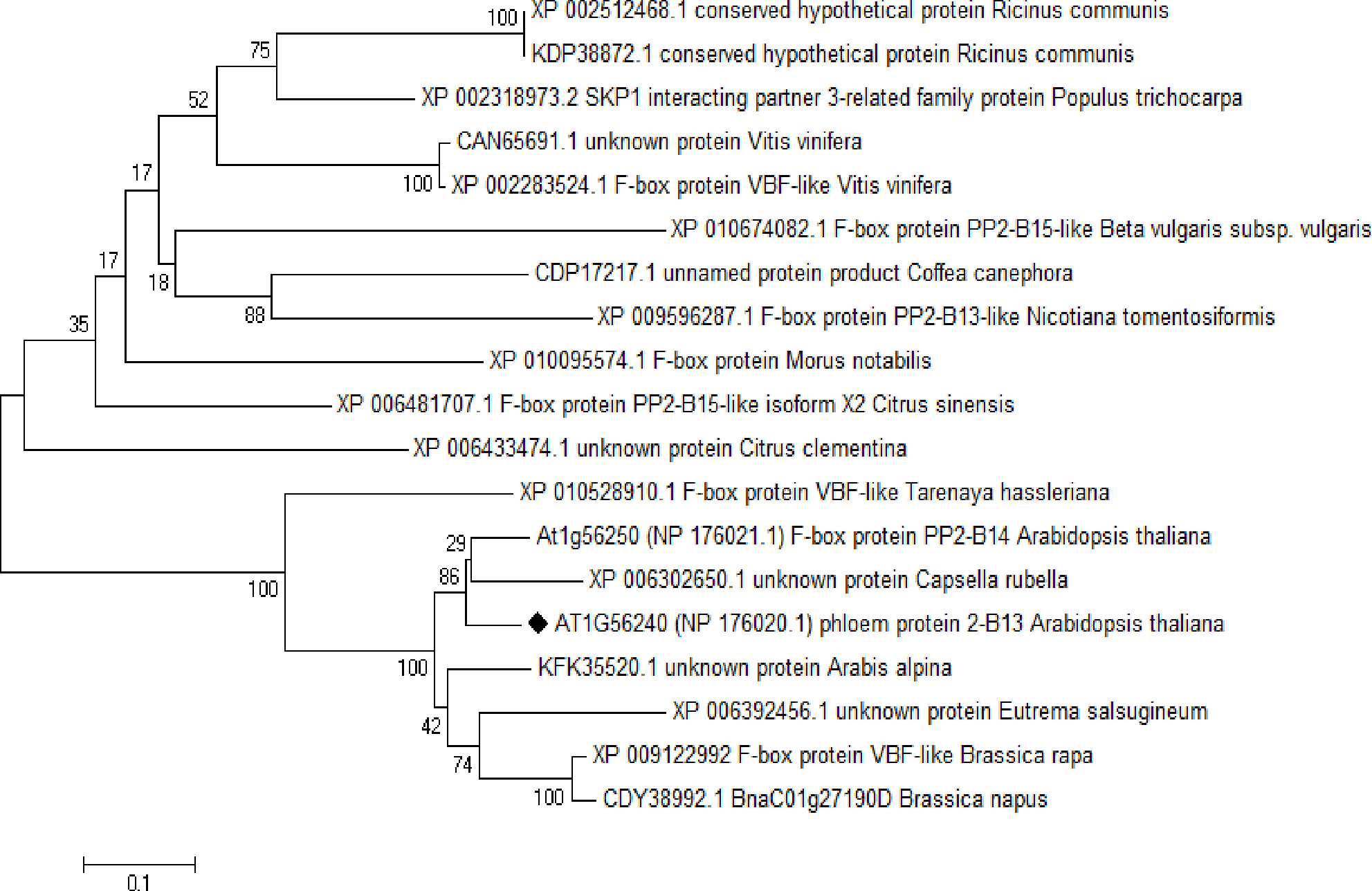

Supplement: S10 Fig — The species indicated are Ricinus communis, Populus trichocarpa, Vitis vinifera, Beta vulgaris, Coffea canephora, Nicotiana tomentosiformis, Morus notabilis, Citrus sinensis, Citrus clementine, Tarenaya hassleriana, Arabidopsis thaliana, Capsella rubella, Arabis alpina, Eutrema salsugineum, Brassica rapa and Brassica napus. PP2-B13 protein in Arabidopsis thaliana was labelled. Sequences for comparisons were obtained from GenBank. The accession numbers and protein names (if available) are given. Analysis was done by maximum likelihood method implemented in MEGA6 (Molecular Evolutionary Genetics Analysis) version 6.0. (TIF) [file pone.0297124.s010.tif]

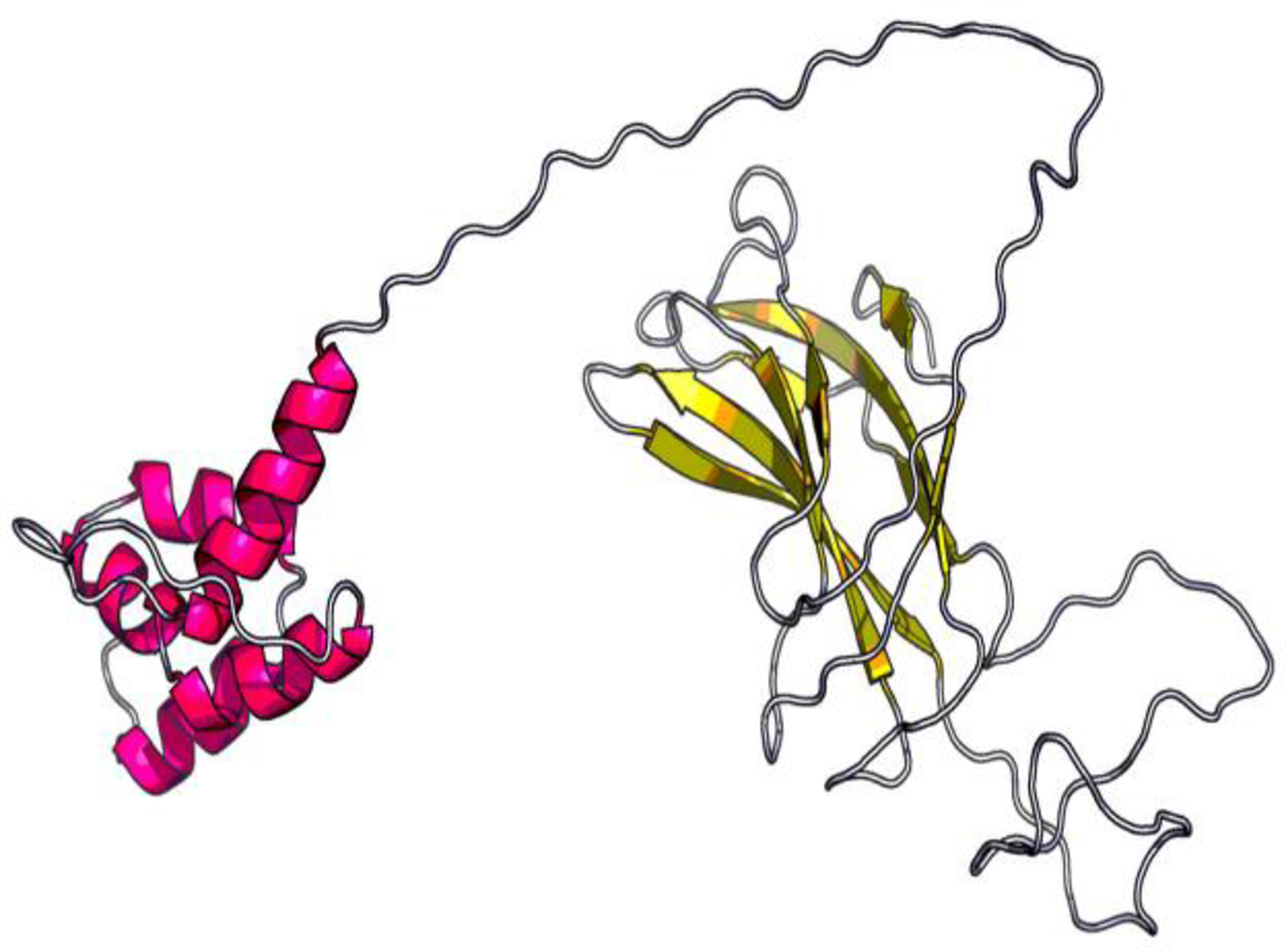

Supplement: S11 Fig — (TIF) [file pone.0297124.s011.tif]

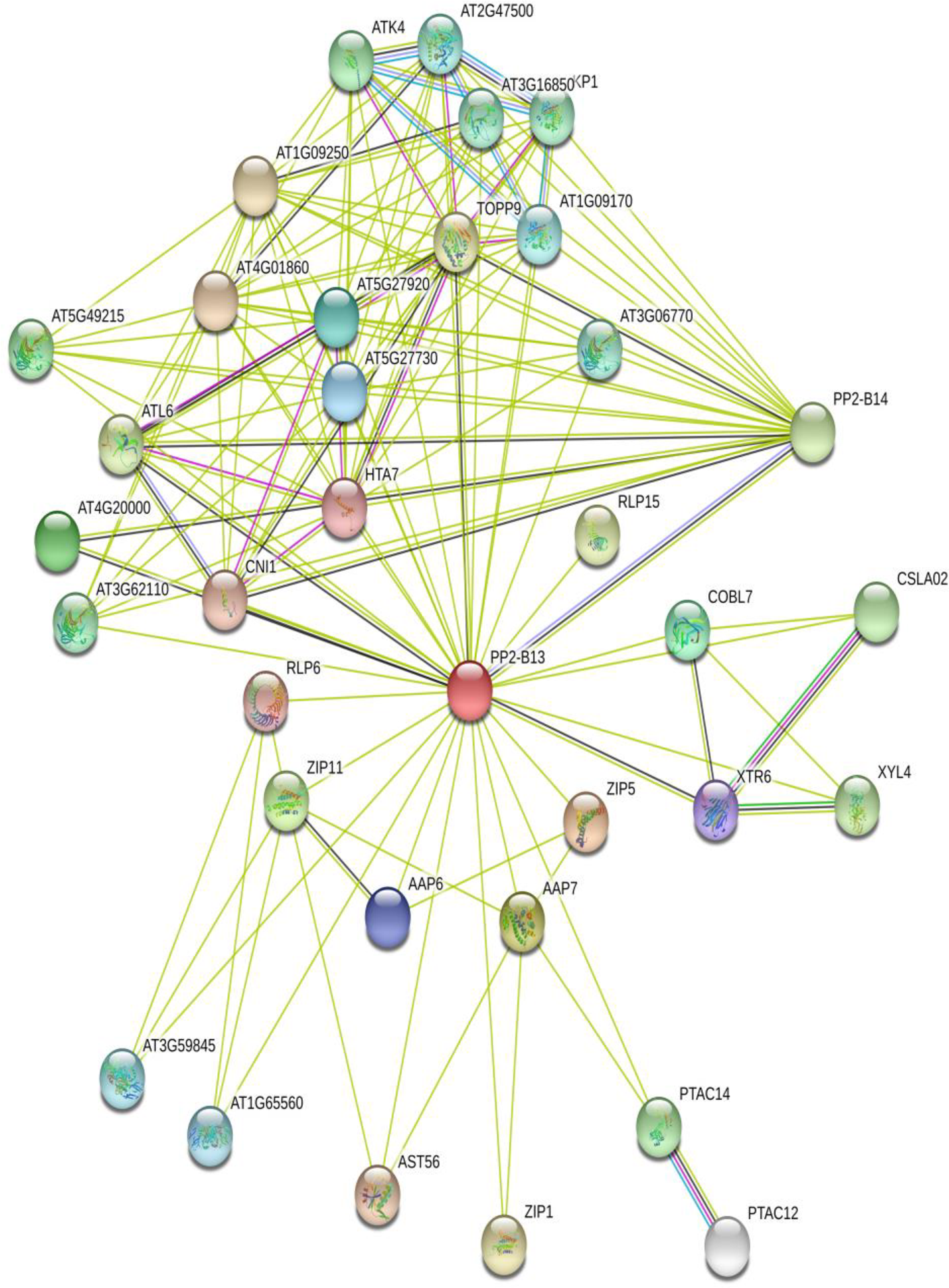

Supplement: S12 Fig — Analysis with a confidence threshold score of 0.4 (Szklarczyk et al. [78]). Line colors indicate the type of interaction used for the predicted associations: gene fusion (red), gene neighborhood (green), co-occurrence across genomes (blue), co-expression (black), experimental (purple), text mining (light green); association in curated databases (light blue). Line thickness represents the strength of data support. Proteins that have a known function in the immune response are marked with dotted lines. (TIF) [file pone.0297124.s012.tif]

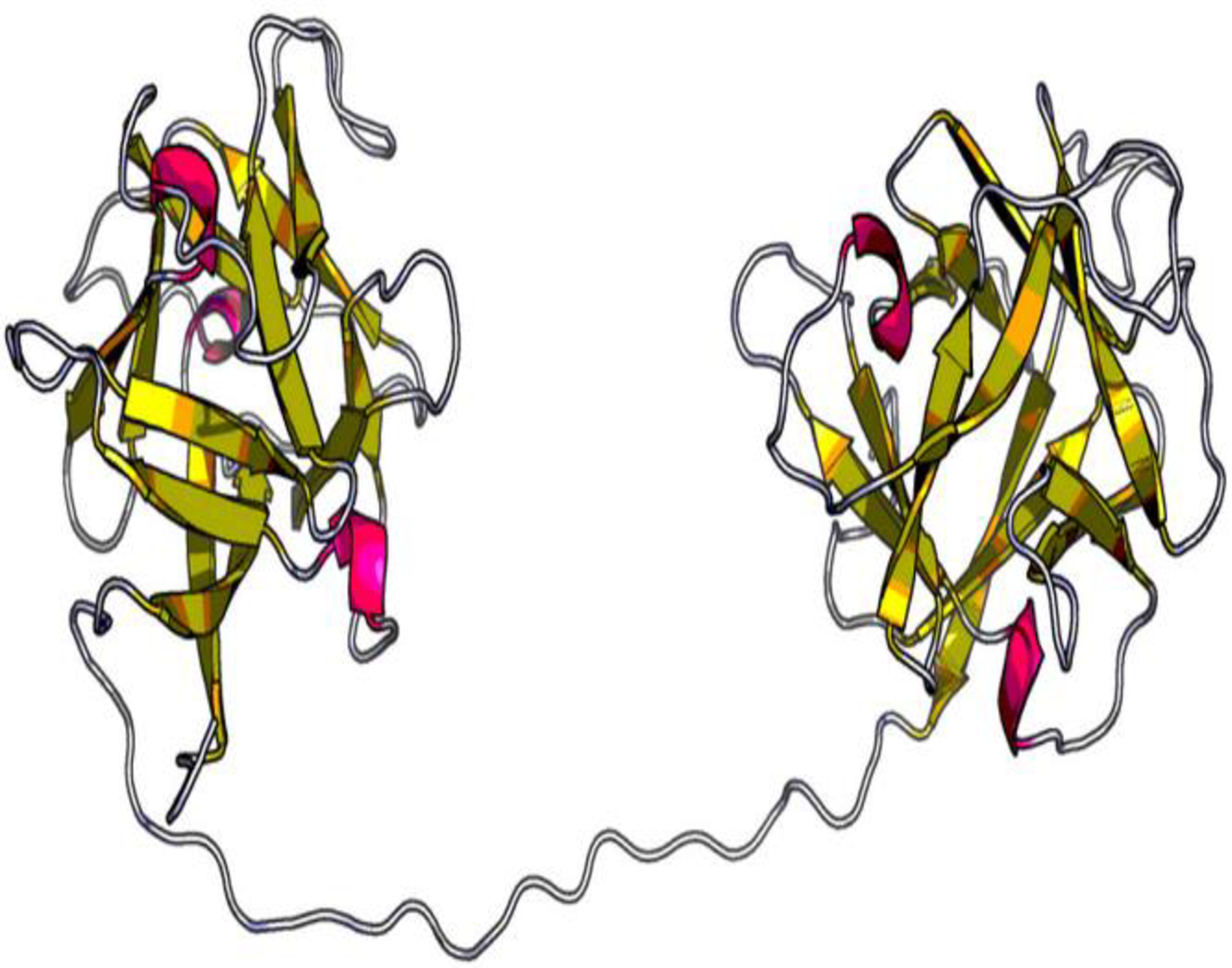

Supplement: S13 Fig — (TIF) [file pone.0297124.s013.tif]

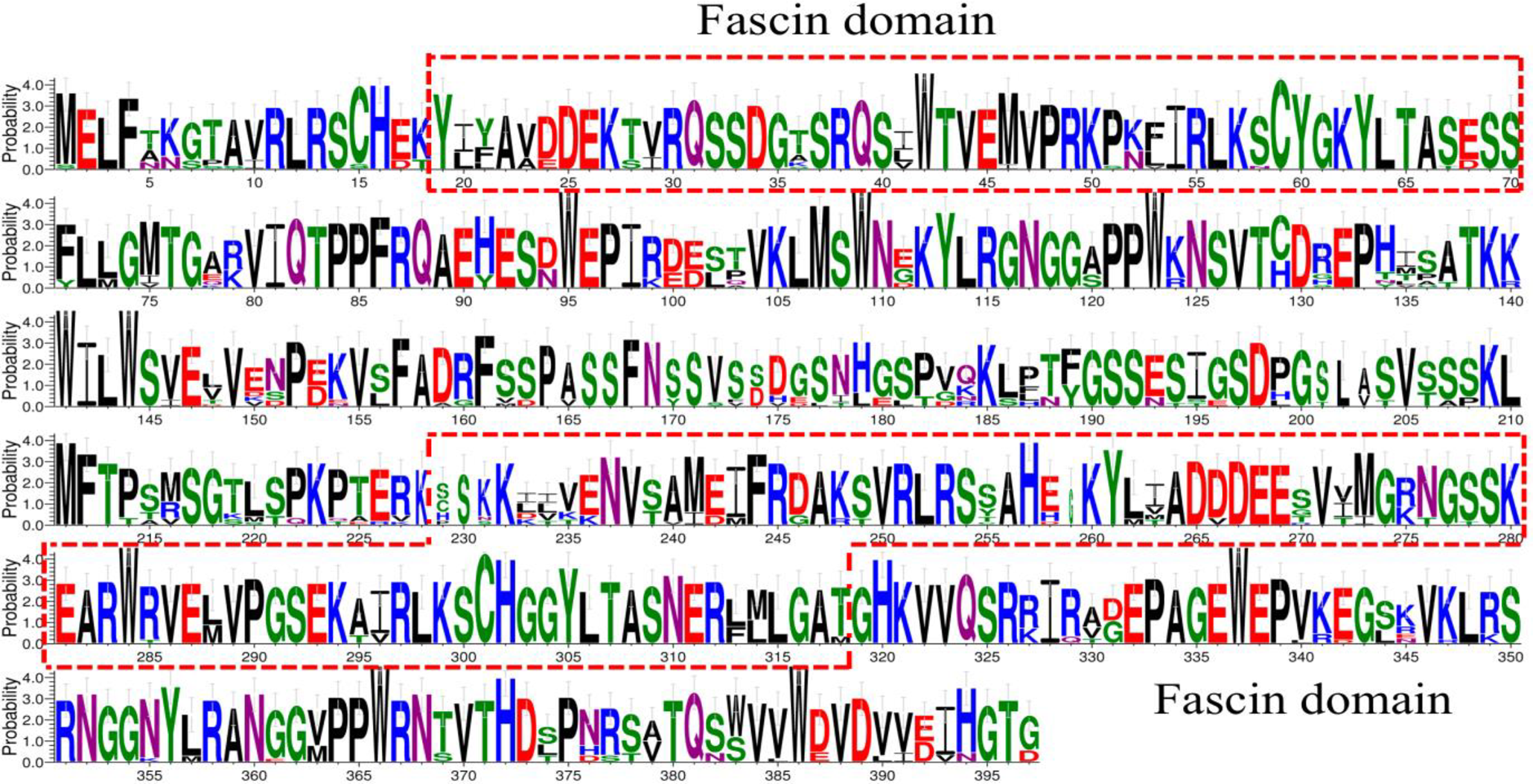

Supplement: S14 Fig — Conservation plots were constructed using WEBLOGO. The y-axis represents the probability score. Y = 4 corresponds to 100% conservation. The predicted domains are highlighted in red boxes. (TIF) [file pone.0297124.s014.tif]

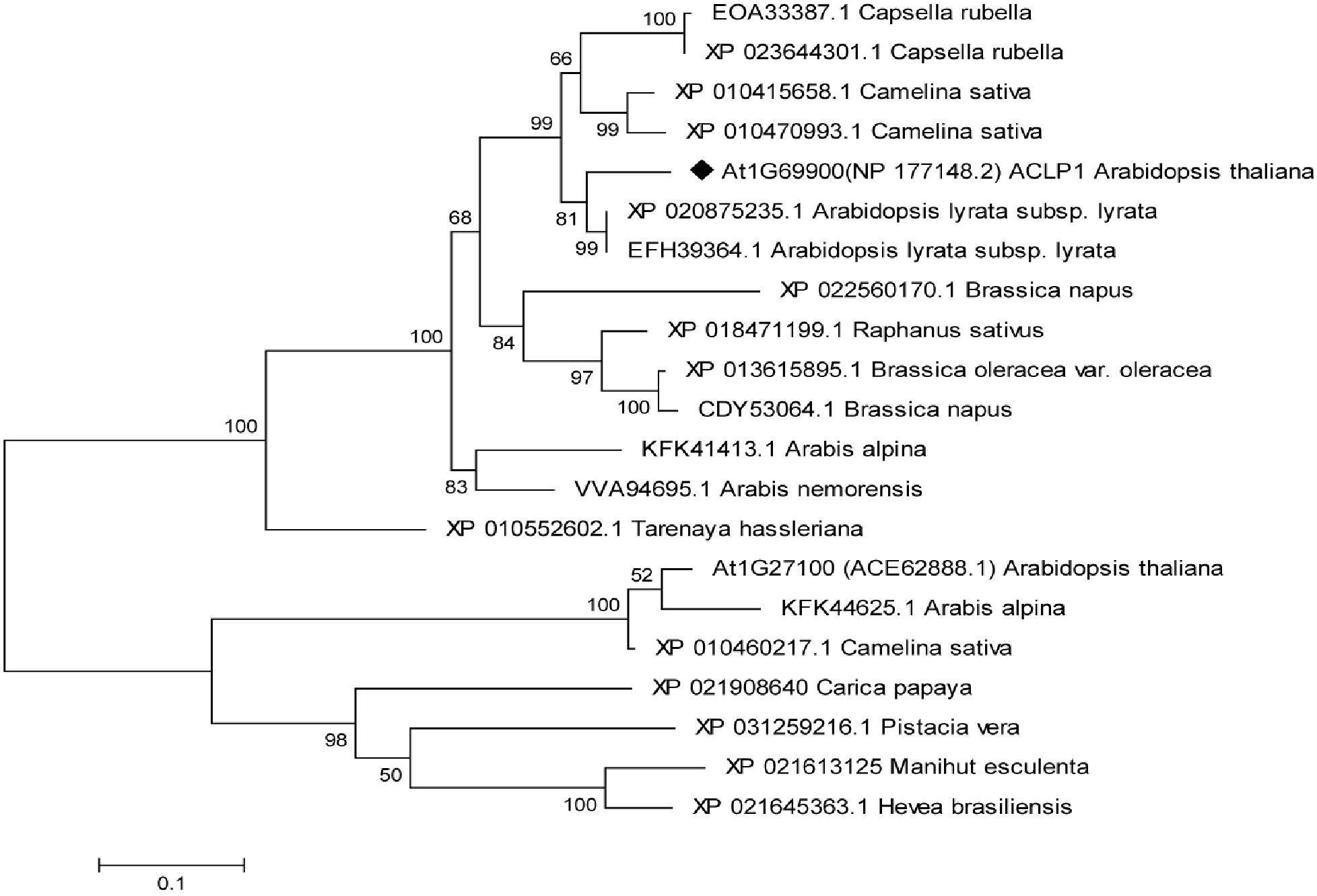

Supplement: S15 Fig — The species indicated are Capsella rubella, Camelina sativa, Arabidopsis thaliana, Arabidopsis lyrata, Brassica napus, Raphanus sativus, Brassica oleracea, Arabis alpina, Arabis nemorensis, Tarenaya hassleriana, Carica papaya, Pistacia vera, Manihut esculenta and Hevea brasiliensis. ACLP1 protein in Arabidopsis thaliana was labeled. Sequences for comparisons were obtained from GenBank. The accession numbers and protein names (if available) are given. Analysis was done by the maximum likelihood method implemented in MEGA6 (Molecular Evolutionary Genetics Analysis) version 6.0. (TIF) [file pone.0297124.s015.tif]

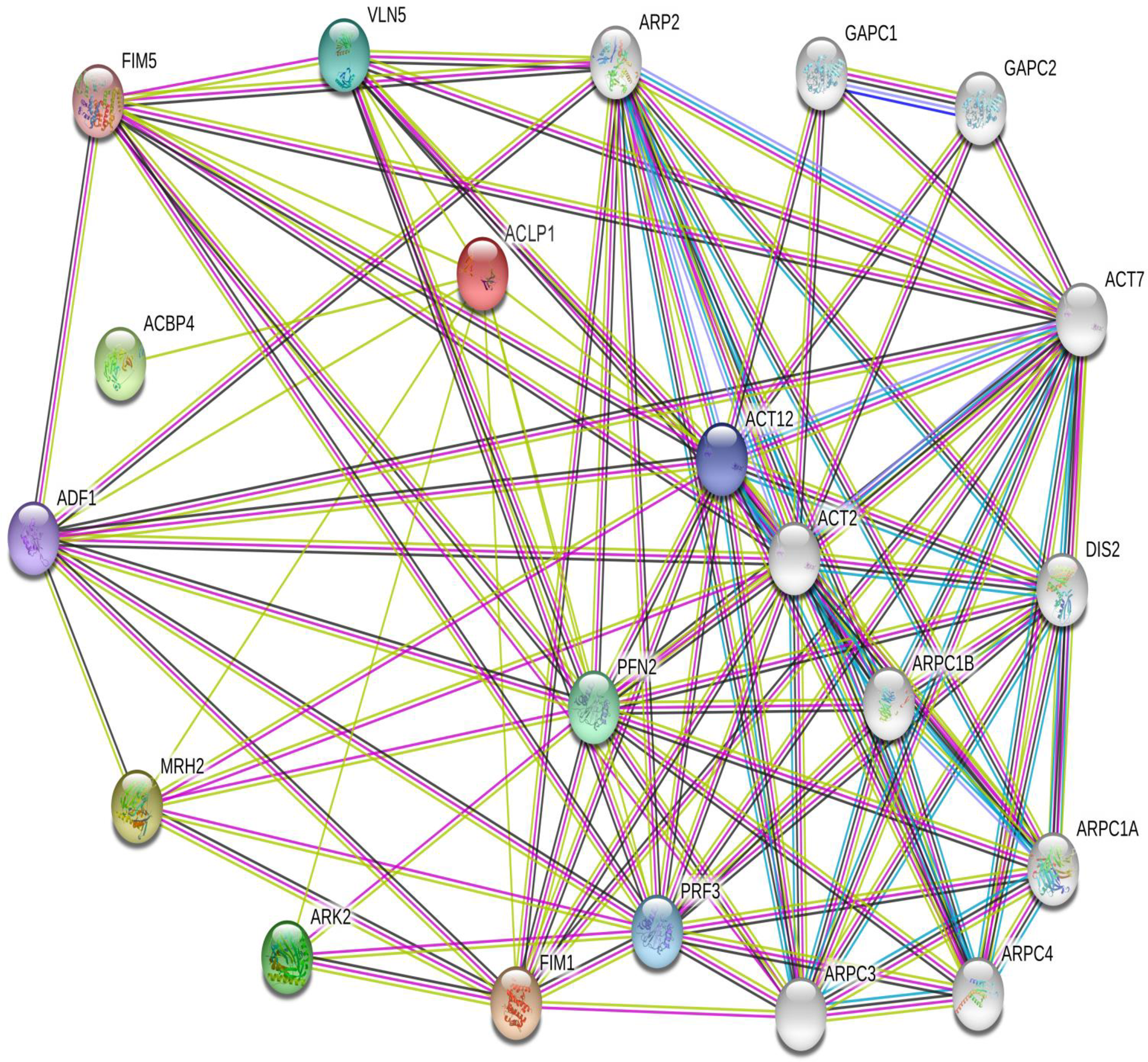

Supplement: S16 Fig — Analysis with a confidence threshold score of 0.4 (Szklarczyk et al. [78]). Line colors indicate the type of interaction used for the predicted associations: gene fusion (red), gene neighborhood (green), co-occurrence across genomes (blue), co-expression (black), experimental (purple), text mining (light green); association in curated databases (light blue). Line thickness represents the strength of data support. Proteins that have a known function in the immune response are marked with dotted lines. (TIF) [file pone.0297124.s016.tif]

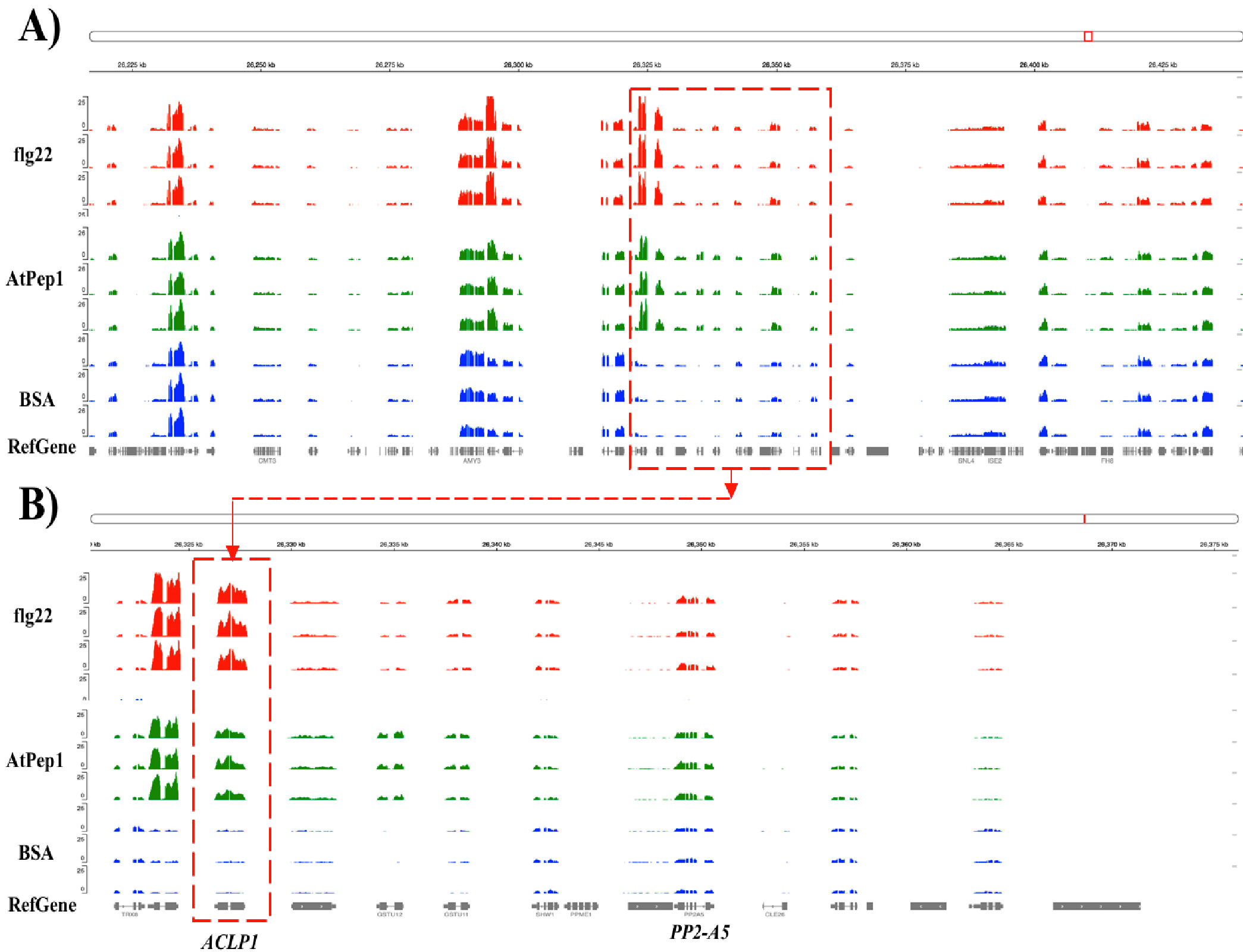

Supplement: S17 Fig — (A) Overlaid depth graphs. (B) Zoomed in view of A. In the graph ACLP1 and PP2-A5 genes are illustrated. (TIF) [file pone.0297124.s017.tif]

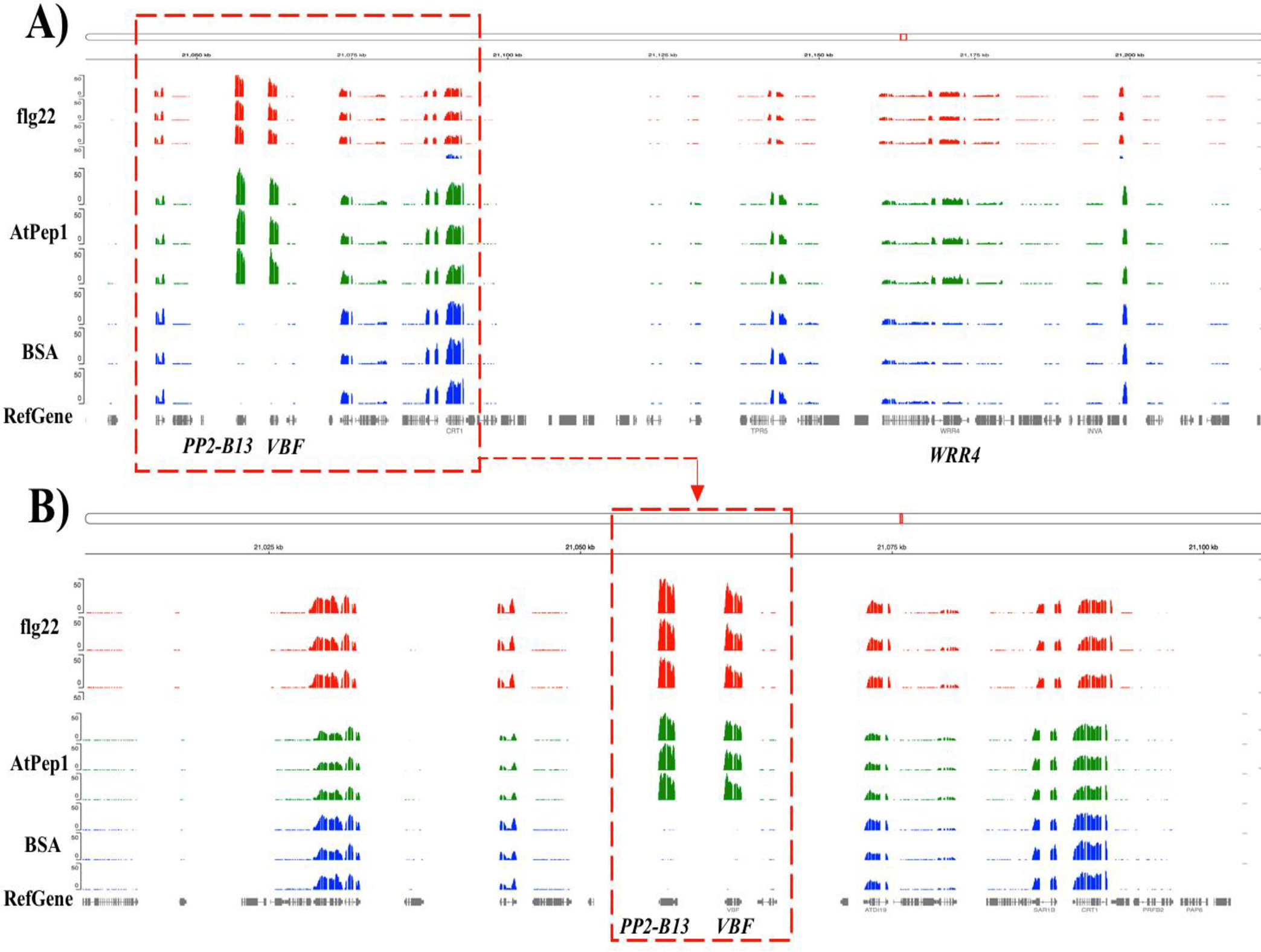

Supplement: S18 Fig — Coverage depth graphs represent transcript abundance. (A) Overlaid depth graphs. (B) Zoomed in view of A. In the graph PP2-B13, VBF and WRR4 genes are illustrated. (TIF) [file pone.0297124.s018.tif]
