# Supplementary material for: Comparative RNA-seq analysis of Arabidopsis thaliana response to AtPep1 and flg22, reveals the identification of PP2-B13 and ACLP1 as new members in pattern-triggered immunity
Source: PLoS One. 2024 Jun 4;19(6):e0297124. doi: 10.1371/journal.pone.0297124 (PMC11149889; doi:10.1371/journal.pone.0297124)
Supplement: S1 Raw images — (DOCX) [file pone.0297124.s037.docx]

# Original, uncropped gel image data

# Comparative RNA-seq analysis of *Arabidopsis thaliana* response to *At*Pep1 and flg22, reveals the identification of PP2-B13 and ACLP1 as new members in pattern-triggered immunity

# Short title: Transcriptomic profiling uncovers novel players in innate immunity in *Arabidopsis thaliana*

**Mehdi Safaeizadeh ^1,2^*, Thomas Boller ^2^, Claude Becker ^3^**

^1^Department of Cellular and Molecular Biology, Faculty of Life Sciences and Biotechnology, Shahid Beheshti University, Tehran, Iran; ^2^Zürich-Basel Plant Science Center, Department of Environmental Sciences, University of Basel, 4056 Basel, Switzerland; ^3^Genetics, LMU Biocentre, Faculty of Biology, Ludwig-Maximilian-University Munich, 82152 Martinsried, Germany

*** Corresponding author: Mehdi Safaeizadeh**

[**Mehdi.Safaeizadeh@unibas.ch**](mailto:Mehdi.Safaeizadeh@unibas.ch)**;**

[**Ma_Safaei@sbu.ac.ir**](mailto:Ma_Safaei@sbu.ac.ir)**;**

[**Mehdi.Safaeizadeh@gmail.com**](mailto:Mehdi.Safaeizadeh@gmail.com)


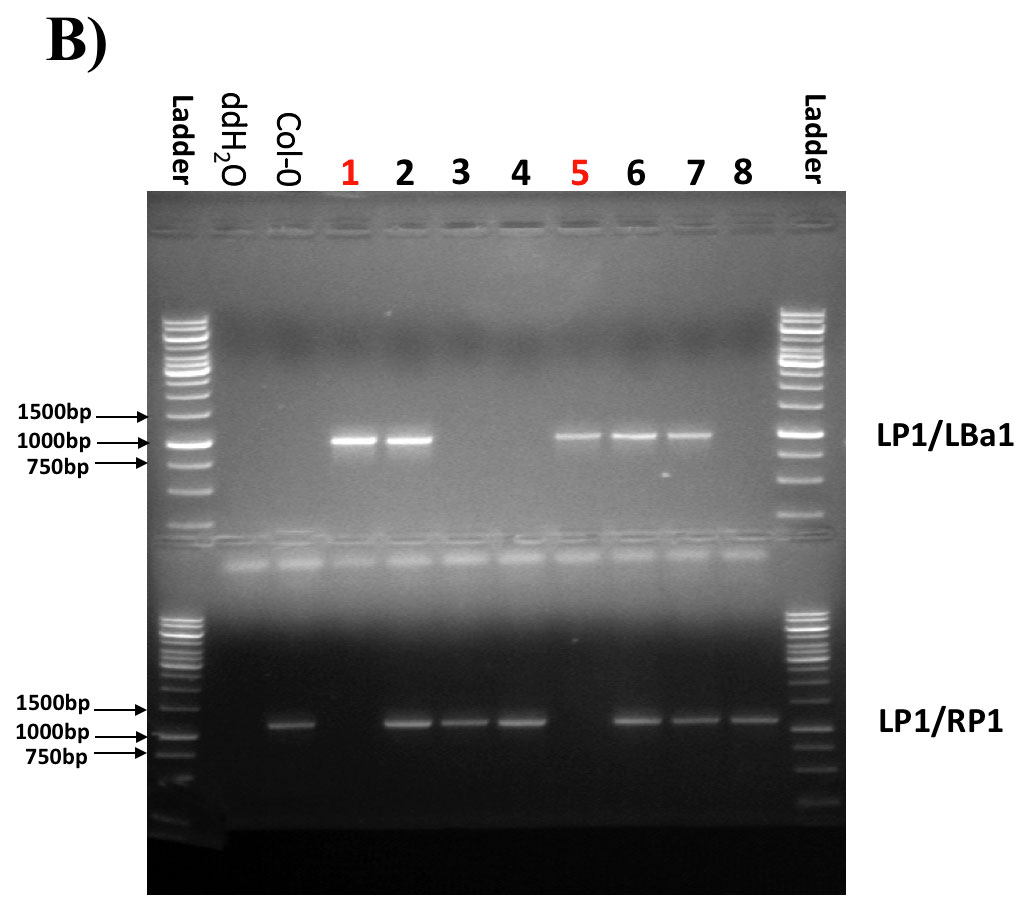


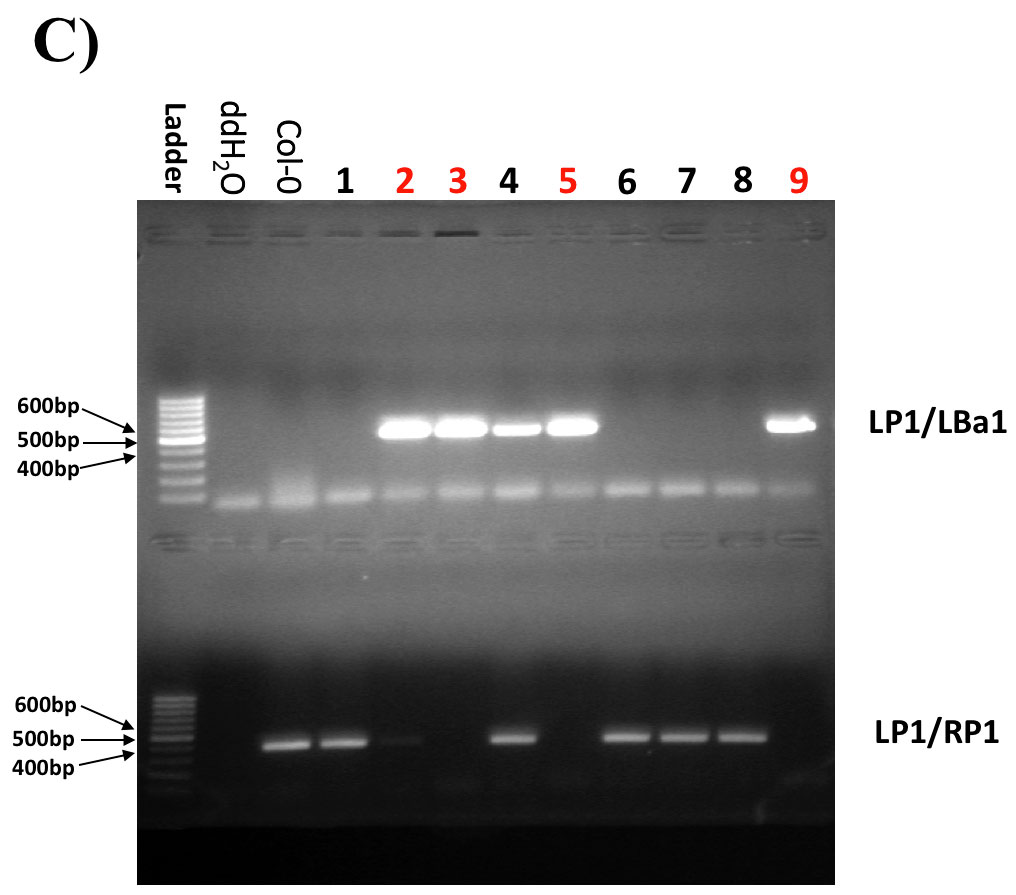


**S2 Fig.**


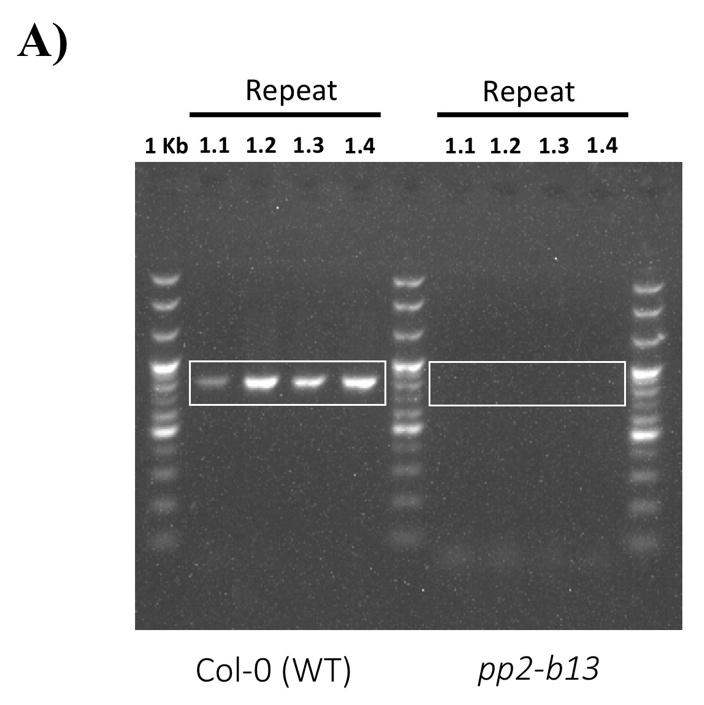

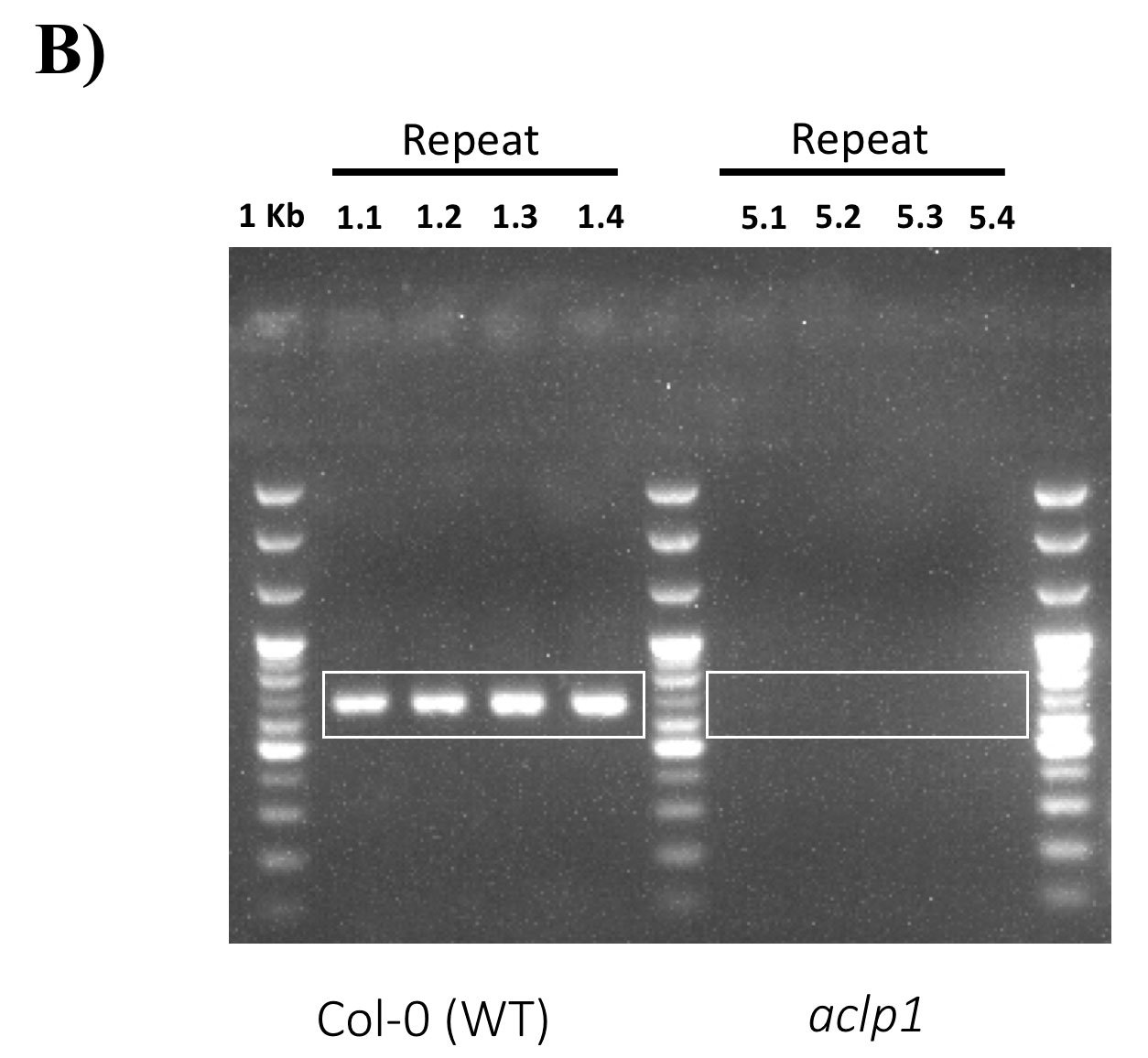

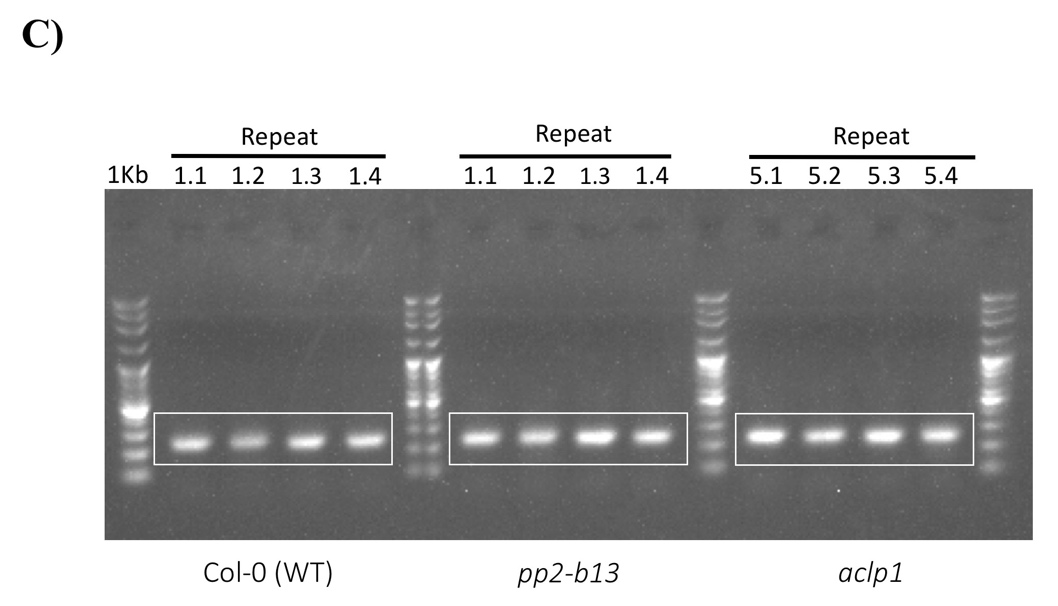


**S3 Fig.**


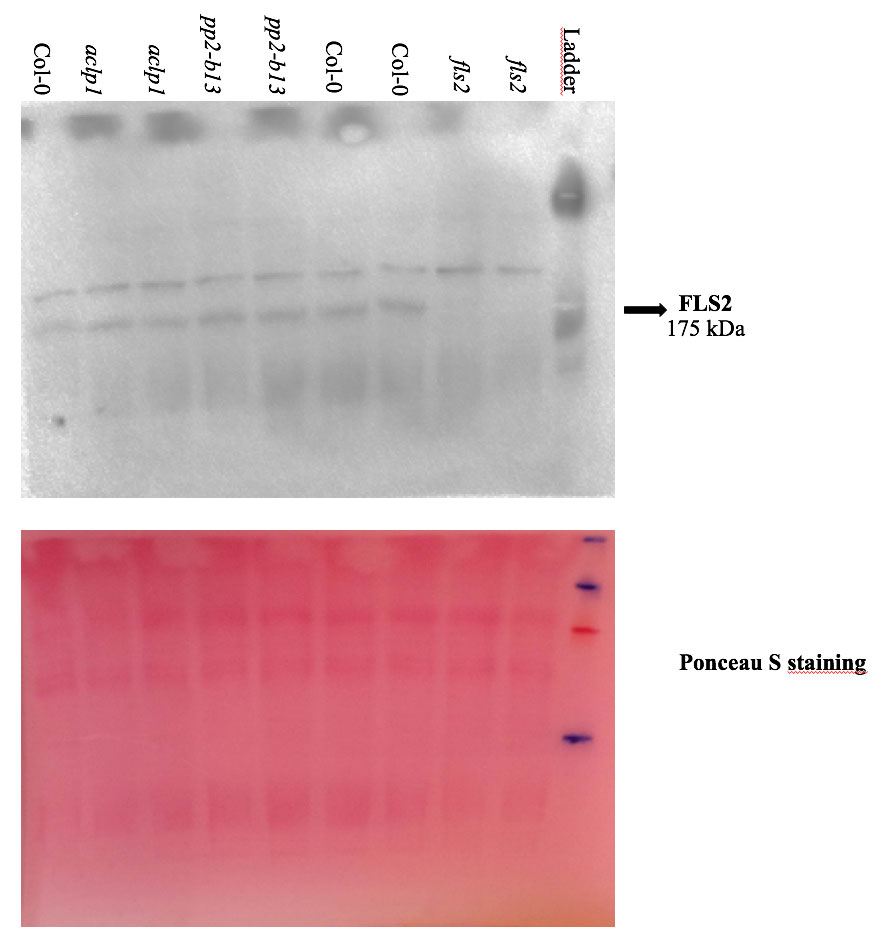


**S8 Fig.**
